# Chromatin landscape in paired human visceral and subcutaneous adipose tissue and its impact on clinical variables in obesity

**DOI:** 10.1016/j.ebiom.2025.105653

**Published:** 2025-03-20

**Authors:** Sadia Saeed, Lars la Cour Poulsen, Tina Visnovska, Anne Hoffmann, Adhideb Ghosh, Christian Wolfrum, Torunn Rønningen, Mai Britt Dahl, Junbai Wang, Akin Cayir, Tom Mala, Jon A. Kristinsson, Marius Svanevik, Jøran Hjelmesæth, Jens Kristoffer Hertel, Matthias Blüher, Tone Gretland Valderhaug, Yvonne Böttcher

**Affiliations:** a Department of Clinical Molecular Biology, EpiGen, Institute of Clinical Medicine, University of Oslo, Oslo, Norway; b EpiGen, Medical Division, Akershus University Hospital, Lørenskog, Norway; c Helmholtz Institute for Metabolic, Obesity and Vascular Research (HI-MAG) of the Helmholtz Zentrum München at the University of Leipzig and University Hospital, Leipzig, Germany; d Laboratory of Translational Nutrition Biology, Institute of Food, Nutrition and Health, ETH Zürich, Schwerzenbach, Switzerland; e Department of Endocrinology, Morbid Obesity and Preventive Medicine, Oslo University Hospital, Oslo, Norway; f Department of Endocrinology, Obesity and Nutrition, Vestfold Hospital Trust, Tønsberg, Norway; g Department of Medicine, University of Leipzig, Leipzig, Germany; h Department of Endocrinology, Akershus University Hospital, Lørenskog, Norway

**Keywords:** Obesity, Adipose tissue, Chromatin accessibility, Gene expression

## Abstract

**Background:**

Obesity is a global health challenge and adipose tissue exhibits distinct depot-specific characteristics impacting differentially on the risk of metabolic comorbidities.

**Methods:**

Here, we integrate chromatin accessibility (ATAC-seq) and gene expression (RNA-seq) data from intra-individually paired human subcutaneous (SAT) and omental visceral adipose tissue (OVAT) samples to unveil depot-specific regulatory mechanisms.

**Findings:**

We identified twice as many depot-specific differentially accessible regions (DARs) in OVAT compared to SAT. SAT-specific regions showed enrichment for adipose tissue enhancers involving genes controlling extracellular matrix organization and metabolic processes. In contrast, OVAT-specific regions showed enrichment in promoters linked to genes associated with cardiomyopathies. Moreover, OVAT-specific regions were enriched for bivalent transcription start site and repressive chromatin states, suggesting a “lingering” regulatory state. Motif analysis identified *CTCF* and *BACH1* as most significantly enriched motifs in SAT and OVAT-specific DARs, respectively. Distinct gene sets correlated with important clinical variables of obesity, fat distribution measures, as well as insulin, glucose, and lipid metabolism.

**Interpretation:**

We provide an integrated analysis of chromatin accessibility and transcriptional profiles in paired human SAT and OVAT samples, offering new insights into the regulatory landscape of adipose tissue and highlighting depot-specific mechanisms in obesity pathogenesis.

**Funding:**

SS received EU-Scientia postdoctoral Fellowship and project funding from the European Union’s Horizon 2020 Research and Innovation program under the Marie Skłodowska-Curie Grant, (agreement No. 801133). LlCP and TR were supported by Helse Sør-Øst grants to Y.B (ID 2017079, ID 278908). MB received funding from grants from the 10.13039/501100001659DFG (10.13039/501100001659German Research Foundation)—Projekt number 209933838—SFB 1052 (project B1) and by 10.13039/100031584Deutsches Zentrum für Diabetesforschung (DZD, Grant: 82DZD00601).

**Research in context**

**Evidence before this study**

Adipose tissue distribution correlates differentially with metabolic complications. Previous data reported adipose tissue depot-specific DNA methylation along with gene expression. Only a handful of studies exist providing chromatin accessibility data in human adipose tissue and no data were available for intra-individually paired samples of human subcutaneous (SAT) and omental visceral adipose tissue (OVAT) in individuals with obesity.

**Added value of this study**

The current study provides global maps of chromatin accessibility and transcriptomic landscape of paired samples of subcutaneous and omental visceral adipose tissue in individuals with obesity. We observed a depot-specific unique chromatin accessibility landscape with about twice as many open chromatin regions in omental visceral adipose tissue. Consistent with chromatin accessibility we also identified an increased number of upregulated genes in OVAT when compared with SAT. We found enrichment for enhancer elements in open chromatin regions in SAT, whilst proximal promoters showed higher chromatin accessibility in OVAT. Related to the dynamic accessible chromatin regions we identified unique transcriptomic patterns associated with clinical variables important to fat distribution and metabolism.

**Implications of all the available evidence**

Our data highlights that chromatin accessibility discriminates subcutaneous from omental visceral adipose tissue and translates into depot-specific transcriptome that correlates with clinically important features. Moreover, we provide a rich compendium of depot specific *cis*-regulatory regions and dynamically expressed genes, representing an extensive resource for further studies of these chromatin elements and associated genes to explore their potential involvement in the pathogenesis of obesity, fat distribution and its related metabolic comorbidities.

## Introduction

Obesity is one of the most challenging health burdens worldwide increasing the risk for metabolic comorbidities including cardiovascular diseases, type 2 diabetes and dyslipidaemia as well as other diseases such as certain types of cancer.^1^ Individual fat distribution rather than overall fat mass correlates with cardiovascular and metabolic disease risk. It is well known that fat storage in human omental visceral adipose tissue (OVAT) confers higher metabolic risk compared with fat deposition in subcutaneous adipose tissue (SAT).^1^, ^2^, ^3^, ^4^, ^5^ We and others have shown evidence for distinct transcriptomic and epigenetic profiles corresponding to different adipose tissue depots, especially in intra-individually paired samples of SAT and OVAT independent of BMI.^6^, ^7^, ^8^, ^9^, ^10^, ^11^, ^12^, ^13^, ^14^ These studies imply a potential adipose tissue depot-specific chromatin architecture along with differential chromatin accessibility and, thereby epigenetic plasticity that may correlate with the observed transcriptional differences and clinical variables related to obesity.

Chromatin accessibility is a hallmark of nuclear hotspots participating in transcriptional regulation and other nuclear processes with a strong enrichment for transcription factor binding sites.^15^^,^^16^ The Assay for Transposase Accessible Chromatin using sequencing (ATAC-seq) with improved omni-ATAC protocol is suggested to provide information-rich chromatin accessibility profiles from technically challenging as well as frozen tissues.^17^ Recent studies involving single-cell and spatial transcriptomics have advanced our understanding of adipose tissue,^18^, ^19^, ^20^ however, its lipid-rich, heterogeneous nature and the technical challenges of chromatin assays have led to limited studies on chromatin accessibility in human,^21^, ^22^, ^23^ mouse,^24^, ^25^, ^26^, ^27^ and porcine^28^^,^^29^ adipose tissue. Previous studies focussing on human adipose tissue and its chromatin accessibility have provided important links between genetic risk variants for cardiometabolic traits located in the accessible regulatory regions and altering adipose tissue function.^21^, ^22^, ^23^ Moreover, mice studies have provided an important resource of accessible regulatory epigenome in mouse adipose tissue.^24^, ^25^, ^26^ A recent study in porcine has mapped chromatin accessibility in two adipose tissue depot-derived stromal vascular fractions (SVFs) using DNase-sequencing (DNase-seq), thus providing insights into distinct chromatin features.^28^ This study is providing a detailed report on integrated analysis of chromatin accessibility and transcriptional profiles in intra-individually paired human samples of subcutaneous and visceral adipose tissue in people with obesity.

In the current study, we hypothesized that potentially distinct open chromatin landscapes in subcutaneous versus visceral adipose tissue depots translate into tissue-specific genome-wide transcriptome profiles. We further sought to investigate whether such depot-specific signatures correlate differentially with relevant clinical variables for obesity, fat distribution, and metabolic traits. To this end, we applied genome-wide ATAC-seq and RNA-seq and generated chromatin accessibility maps and expression profiles in paired samples of subcutaneous and omental visceral adipose tissue biopsies from people with obesity. Here, we show differentially accessible *cis-*regulatory open chromatin regions discriminating the two adipose tissue depots. In addition, we determined transcription factors (TFs) binding motifs in both depots that were highly enriched in differentially accessible regions. We further substantiated the functional relevance of the identified *cis-*regulatory elements and the associated genes by showing an enrichment for highly relevant functional pathways and gene ontology terms as well as their correlation with important clinical variables of obesity.

## Methods

### Study design of the discovery cohort

Adipose tissue biopsies were obtained from SAT and OVAT during initial phase of bariatric surgery (eight patients with obesity, BMI ≥ 35 kg/m^2^). Individual samples marked with ‘A’ were obtained from Oslo University Hospital, Aker, Oslo, Norway, whereas sample marked with ‘X’ was obtained from Vestfold Hospital Trust, Tønsberg, Norway. For accessibility profiling fresh biopsies were transported on ice and immediately processed for nuclei isolation. Whereas, for transcriptomic analyses biopsies were immediately snap frozen on dry ice/in liquid nitrogen to prevent degradation and stored at −80° C until further processing. All study protocols have been approved by the Regional Ethics Committee in Health Region South-East Norway (2017/1528). All participants gave written informed consent before taking part in the study. Main characteristics of the study population are summarized in Table 1.

**Table 1:** Main clinical characteristics of the individuals involved in the study.

Clinical characteristics	N	Mean	SD
Age, years	8	36.00	±8
Male/Female	0/8	–	–
Waist, cm	8	114.8	±13
Hip, cm	7	130.3	±14.1
WHR	7	0.88	±0.06
BMI, kg/m2	8	43.8	±7.1
Fasting serum glucose, mmol/L	8	6.4	±1.96
Total Cholesterol, mmol/L	8	4.3	±1.1
HDL cholesterol, mmol/L	8	1.2	±0.2
LDL cholesterol, mmol/L	8	2.4	±0.8
Triglycerides, mmol/L	8	1.4	±0.5
Fasting serum insulin, pmol/L	7	203.6	±154.6
HOMA_IR, mmol/l∗pmol/L/135	7	9.18	±5.7
HbA1c, mmol/mol	8	43.6	±14.8
T2D yes/no	2/6	–	–

The results are shown as means and ± standard deviation (SD).

### Study designs of the validation cohorts

The human data used for the validation cohort were sourced from the Leipzig Obesity Biobank (LOBB; https://www.helmholtz-munich.de/en/hi-mag/cohort/leipzig-obesity-bio-bank-lobb), which includes paired samples of abdominal SAT and OVAT, along with body fluids and associated anthropometric data. Adipose tissue samples were collected during elective laparoscopic abdominal surgeries, following established protocols.^30^ Body composition and metabolic parameters were assessed using standardized methods as described in previous studies.^31^^,^^32^ Exclusion criteria included participants under 18 years of age, those with chronic substance or alcohol misuse, smokers within the 12 months prior to surgery, individuals with acute inflammatory diseases, those taking glitazones as concomitant medication, patients with end-stage malignant diseases, individuals who experienced weight loss exceeding 3% in the three months leading up to surgery, and those with uncontrolled thyroid disorders or Cushing’s disease.

The cross-sectional cohort (CSC) comprises 1480 individuals, of whom 31 had normal weight or were overweight (53% women; age 56.4 ± 13.3 years; BMI 25.5 ± 2.6 kg/m^2^), while 1449 had obesity (71% women; age 46.9 ± 11.7 years; BMI 49.2 ± 8.3 kg/m^2^).

The metabolically healthy and unhealthy obesity cohort (MHO/MUO; N = 73) consists of 31 insulin-sensitive (IS) (71% women; age 38.8 ± 11.1 years; BMI 45.9 ± 6.9 kg/m^2^; fasting plasma glucose (FPG): 5.2 ± 0.2 mmol/l; fasting plasma insulin (FPI): 27.9 ± 13.5 pmol/l) and 42 insulin-resistant (IR) individuals (71% women; 47.2 ± 7.7 years; BMI 47.3 ± 8.1 kg/m^2^; FPG: 5.7 ± 0.3 mmol/l; FPI: 113.7 ± 45.7 pmol/l).

The bariatric surgery cohort (BSC) consists of 65 individuals with morbid obesity (66% women) who underwent bariatric surgery for weight loss. The surgical procedures typically began with laparoscopic sleeve gastrectomy, followed by laparoscopic Roux-en-Y gastric bypass. The average preoperative BMI and age of the cohort were 54.5 ± 9.3 kg/m^2^ and 44.1 ± 9.2 years, respectively. At the time of the second surgery, patients had an average BMI of 40.9 ± 7.2 kg/m^2^ and an average age of 47.1 ± 9.9 years. On average, patients lost 40.2 ± 21.2 kg between the two surgeries, with only those who lost more than 5 kg included in the BSC. The average time between surgeries was 3.0 ± 3.9 years.

### ATAC-seq library preparation of the discovery cohort

A previously described omni-ATAC protocol^17^ was followed for adipose tissue library preparation. The initial tissue amounts and nuclei isolation volumes were increased 10-fold to obtain a sufficient number of nuclei. Briefly, 200–400 mg of fresh adipose tissue biopsies were finely minced and transferred to 10 mL of cold HB solution in a 15 mL Dounce homogenizer pre-chilled on ice. The tissue was homogenized using Piston A only, until resistance ceased. Floating fat and debris were removed at all steps using cotton swabs. The homogenate was filtered through a 70 μm Flowmi strainer during transfer to pre-chilled 5 mL LoBind tubes. Nuclei were pelleted by centrifugation at 350 RCF for 5 min at 4 °C using a fixed-angle centrifuge. The supernatant was removed, and the nuclei were gently resuspended in 5 mL of HB buffer for washing before centrifuging again. After removing the supernatant, the nuclei were resuspended in a total of 400 μL of HB buffer. The nuclei were then purified on iodixanol gradients by spinning at 3000 g for 1 h, subjected to transposition, and DNA cleanup as described.^17^ Libraries were enriched for 150–250 bp fragments using Pippin Prep (Sage Science) and sequenced as paired end reads (150 cycles) on the HiSeq4000 platform (Illumina) at the Norwegian Sequencing Centre, Oslo University Hospital.

### Processing of ATAC-seq data

Quality control of sequencing data was performed using FastQC^33^ followed by read trimming with Trimmomatic^34^ to remove low quality reads and remnants of the adaptors used. The trimmed reads were mapped to the human genome hg38 with bowtie2^35^ and the mapping reads (bam files) were further processed with picard^36^ and samtools.^37^ Duplicates were marked using Picard, and reads that were mapped to the mitochondrial genome with a mapping quality below 10, as well as reads with SAM flag 1804^36^ were removed from further analysis. ATACseqQC^38^ quality check was performed, and alignmentSieve from deepTools^39^ was used to shift coordinates of the processed bam files to account for the ATAC-seq specifics. The shifted processed reads were used for peak calling with MACS2.^40^ Encode defined anomalies were removed from the called peaks using BEDTools,^41^ peak files were transformed to bigwigs with bedGraphToBigwig.^42^

The narrow peaks from all the samples were pooled together and overlapping peaks as well as the peaks with at most 10 bp distance were merged together using BEDTools.^41^ From the set of merged peaks, the peaks that were present in only one sample were removed. All ATAC-seq sequencing statistics are provided in Supplementary Table S1. Read counting in the accessible regions was performed with subread’s featureCounts,^43^ and principal component analysis (PCA) plots were generated from the read counts. The ATAC-seq data processing pipeline along with an RNA-seq data processing pipeline and a differential analysis pipeline^44^ which are described in detail below, were developed for this project to operate on the national high-performance computing cluster dedicated to handle human sensitive data. The pipelines are orchestrated with Snakemake^45^ and bioinformatics tools are provided as singularity^46^ containers which makes the pipelines easily transferable to other computational resources and the analyses reproducible. We make the ATAC-seq pipeline version 0.1.1 publicly available in the provided gitlab repository and as well as a set of input reference data and configs got persistent DOIs.^47^^,^^48^ This work was performed on the TSD (Tjeneste for Sensitive Data) facilities, owned by the University of Oslo, operated and developed by the TSD service group at the University of Oslo, IT-Department (USIT) (tsd-drift@usit.uio.no).

### Differential accessibility analysis of ATAC-seq data

The read counts generated while processing ATAC-seq data were used to perform differential accessibility analysis with DESeq2.^49^ The differential accessibility analysis was performed in a paired manner - SAT and OVAT samples from the same individual were considered a pair (multi-factor design was used to account for subject and tissue type at the same time). Accessible regions with log_2_(Fold Change) > 1 and FDR adjusted *p*-value < 0.05 were considered differentially more accessible in OVAT when log_2_ Fold Change was positive and differentially more accessible in SAT when log_2_ Fold Change was negative. Volcano plots were generated to visualize logarithms of fold-change (between the tissue types) and FDR adjusted *p*-value (of observing such a fold-change by chance) against each other for every accessible region in the analysed samples. The tabular results generated at this stage were further used in the promoter analysis. The differential accessibility analysis was performed as a part of the FeralFleaPainter pipeline,^44^ which is publicly available at gitlab repository.^50^ Version 0.1.1 of the pipeline (that was used for this analysis) as well as a set of input reference data and configs got persistent DOIs.^50^^,^^51^

### Peak distribution, overlap with other adipose tissue ATAC-seq datasets and roadmap epigenomic chromatin states

ChIPseeker^52^ was used for the genomic annotation of accessible peaks per sample as well as differentially accessible regions. We overlapped the ATAC-seq peaks of human adipose tissue origin from (Canon ME et al.)^22^ and ENCODE ATAC-seq dataset of *Homo sapiens* subcutaneous adipose tissue originating from a female adult (53 years) GSM5258563 using BEDTools. For chromatin states analysis we obtained chromatin states data for adipose nuclei originating from subcutaneous abdominal adipose tissue (E063) based on an 18-state model using six histone marks (H3K4me1, H3K4me3, H3K36me3, H3K27me3, H3K9me3, and H3K27ac) from the Roadmap Epigenomics Consortium.^53^ We calculated the number of differentially accessible regions overlapping with each chromatin state using BEDTools.^41^ For further enrichment analysis related to ENCODE adipose tissue (E063) chromatin states, we compared the differences between observed and expected frequencies using the Fishers’s exact test (two sided). The results are represented as *–*log_10_ *p-*values for enrichment or depletion. The significance levels for *p*-values are as follows: ∗*p* < 0.05, ∗∗*p* < 0.01, ∗∗∗∗*p* < 0.0001.

### Motif analysis

Motif enrichment analysis was performed on differentially accessible regions between SAT and OVAT against JASPAR2022_CORE_vertebrates_non-redundant database^54^ using MEME-suit Simple Enrichment Analysis of motifs (SEA) version 5.5.1 with default settings (expected value (E) ≤ 10).^55^ The enrichment E-value of a motif is its adjusted *p*-value multiplied by the number of motifs in the input. Among the significantly enriched motifs (E ≤ 10), motifs with true positive (TP) values < 10% were manually removed to finally report the motifs with greater than 10% occurrence in respective differentially accessible regions.

### Adipocyte isolation

Primary adipocytes (AC) were isolated from fresh adipose tissue biopsies obtained from a subset of patients (N = 5). Briefly, tissue samples were rinsed, finely minced, and incubated at 37 °C for 45 min with 2 mg/mL collagenase type I (Worthington) in AIS buffer, which contained 5.5 mM D-glucose, 4% BSA, 0.8 mM ZnCl_2_, and 500 nM adenosine in a Krebs-Ringer-bicarbonate-HEPES solution (pH 7.2). After digestion, the tissue was filtered through a 400 μm nylon mesh and centrifuged at 150 g for 8 min to separate adipocytes from the stromal vascular fraction (SVF). The floating adipocyte layer was washed twice in AIS buffer, snap-frozen in liquid nitrogen, and stored at −80 °C for further analysis.

### RNA isolation and RNA-seq library preparation of the discovery cohort

Frozen adipose tissue biopsies and primary adipocytes were lysed directly in Qiazol and homogenized passing the lysate through a 18G needle (adipocytes) or using ceramic beads in a MP BIO FastprepTM 5 G unit with 6.0 m/s for 2∗ 30 s. The homogenized lysate was centrifuged for 5 min, and the floating lipid phase was discarded. Phase separation was performed with chloroform. RNA from the liquid phase was precipitated with 70% EtOH, purified on RNeasy MinElute spin columns and subjected to on-column DNase digestion (RNeasy Micro kit; QIAGEN). RNA integrity was evaluated on Bioanalyzer (Agilent). Samples with RIN < 5 were excluded from further analyses. RNA-seq libraries were generated from 50 ng fragmented RNA using SMARTer Stranded Total RNA-Seq Kit v2 - Pico Input (Takara) according to the protocol for fragmented RNA. Libraries were sequenced as paired ends with 150 cycles, on the HiSeq4000 platform (Illumina) at the Norwegian Sequencing Centre, Oslo University Hospital.

### Processing of RNA-seq data

Initial quality check of raw sequencing data with FastQC^33^ was followed by read trimming with Trimmomatic^34^ to remove low quality parts of the reads and remnants of the used adaptors. The trimmed reads were mapped to human genome hg38 with STAR^56^ and the mapping reads (bam files) were further processed with samtools^37^ - only read pairs mapping properly as pairs to the reference genome were considered for further analysis and reads with mapping quality below 10 and reads with sam flag 1548^36^ were removed from the further analysis. Processed bam files were transformed to bigwigs with bamCoverage from deepTools.^39^ Afterwards, read counting using GENCODE gene annotation v31 was performed with subread’s featureCounts,^43^ the read counts were normalized to transcripts per million (TPM).^57^ The RNA-seq data processing pipeline^44^ is publicly available at gitlab repository,^58^ version 0.1 (that was used for this analysis) as well as a set of input reference data and configs got persistent DOIs.^58^^,^^59^

### Differential expression analysis of RNA-seq data

The read counts generated while processing RNA-seq data were used to perform differential expression analysis with DESeq2.^49^ The differential expression analysis was performed with similar sample and threshold settings as described for differential accessibility analysis log_2_(Fold Change) > 1 and FDR adjusted *p-*value < 0.05 in a paired manner - SAT and OVAT samples from the same individual were considered a pair (multi-factor design was used to account for subject and tissue type at the same time).

Volcano plots were generated to visualize logarithms of fold-change (between the tissue types) and FDR adjusted *p*-value (of observing such a fold-change by chance) against each other for every gene expressed in the analysed samples. The tabular results generated at this stage were further used in the promoter analysis. The differential expression analysis was performed as a part of the FeralFleaPainter pipeline,^44^ with details as described above for differential ATAC-seq analysis.

### Overlap of differentially accessible regions with gene expression in promoter regions

For each gene analysed in differential expression analysis between OVAT and SAT, all open chromatin regions (OCRs) located within 1000 bp up- and downstream of the transcription start site of the gene were classified into twelve different categories showed in Supplementary Table S9. From these 12 collections group 3, 7 and 11 were excluded from further analyses due to very few regions to no regions enrichment. Categorization of the genes and their respective nearby OCRs was performed as a part of the FeralFleaPainer pipeline.^44^

### Super enhancer analysis

Adipose tissue related super enhancer dataset was obtained from Super Enhancer database SEdb 2.0 adipose tissue_hg38.^60^ BEDTools were used to compute overlap of the differentially accessible regions with adipose tissue super enhancers. Genes annotated with respect to overlapping differentially accessible regions (ChIPseeker annotation of current study DARs) and genes annotated in close proximity to over lapping super enhancers by SEdb^60^ were used for further analysis. Above-mentioned genes that showed corresponding overlap of a super enhancer and differentially accessible regions were further filtered based on their differential expression log_2_(Fold Change) > 1 and FDR adjusted *p-*value < 0.05 and those genes up regulated in SAT were further used for Gene Ontology (GO) terms and pathway analysis.

### Gene ontology and pathway analysis

All the pathway and gene ontology analyses were performed using enrichr^61^(https://maayanlab.cloud/Enrichr/) and ShinyGO version 0.76.3 with default parameters.^62^

### Library preparation and RNA-seq analysis of the validation cohort

The library preparation and RNA-seq data processing were conducted as previously described.^63^ Briefly, RNA was extracted from adipose tissue (AT) samples using the SMARTseq protocol.^64^ Single-end sequencing of the libraries was performed on a NovaSeq 6000 instrument at the Functional Genomics Centre in Zurich. Raw sequencing reads underwent adaptor and quality trimming using Fastp^65^ (v0.20.0). Read alignment to the human reference genome (assembly GRCh38.p13, GENCODE release 32)^66^ and gene-level expression quantification were carried out using Kallisto^67^ (v0.48). Samples with read counts exceeding 20 million were downsampled to 20 million using the R package ezRun (v3.14.1; https://github.com/uzh/ezRun, accessed on 23 March 2022). The data were normalized using a weighted trimmed mean (TMM) of the log expression ratios and adjusted for age and sex.

### Statistical analysis

Spearman correlation of the clinical variables with gene expression (transcripts per million, TPM) values along with hierarchical clustering was performed using Hmisc v5.2-2, corrplot v0.95^68^ and pheatmap v1.0.12 packages in software R v4.2.3. All correlations with a *p*-value less than 0.05 were considered significant. For the validation cohorts between-group comparisons were performed using a non-parametric statistical aproach with the R package ggstatsplot^69^ v0.10.1, based on one-way Kruskal–Wallis ANOVA and pairwise Mann–Whitney U test, corrected for multiple comparisons using the Hommel method.

### Visualization of the data

All the visualization plots were generated using GraphPad prism6 and R v4.2.3. Genome wide data was visualized using the Integrative Genomic Viewer (IGV) browser.^70^

### Ethics

All study protocols have been approved by the Regional Ethics Committee in Health Region South-East Norway (2017/1528). The LOBB study was approved by the Ethics Committee of the University of Leipzig (approval no: 159-12-21052012) and performed in accordance with the Declaration of Helsinki. All participants gave written informed consent before taking part in the study.

### Role of funders

Funders for the current study were not directly involved in the design, data collection, analysis, interpretation or writing of this manuscript.

## Results

### Chromatin accessibility landscapes of SAT and OVAT show clear depot-specificity

**Fig. 1:**
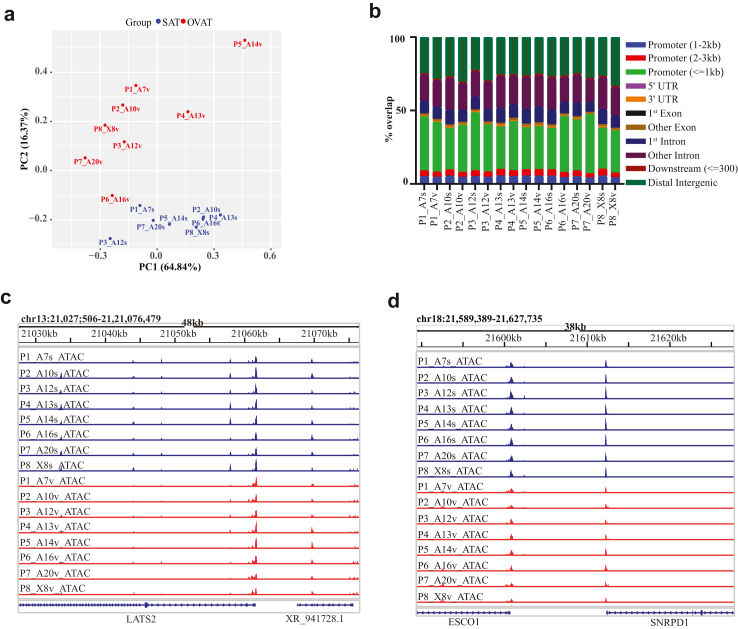
**Chromatin accessibility mapping (ATAC-seq) in intra-individually paired SAT and OVAT samples from patient’s with obesity.** a) Principal component analysis performed on read counts from all SAT and OVAT samples. b) Genomic distribution of ATAC-seq called accessibility peaks in all SAT (P1s-P8s) and OVAT (P1v-P8v) samples. c and d) IGV browser view of the ATAC-seq signals of two different exemplary regions in all the subcutaneous (blue) and visceral (red) adipose tissue samples. Abbreviations: SAT = subcutaneous adipose tissue, OVAT = omental visceral adipose tissue.

To characterize the chromatin accessibility landscape, we analysed the open chromatin regions generated by ATAC-seq from the human adipose tissue samples of SAT and OVAT (Supplementary Table S1). Principal component analysis of all ATAC-seq data from both depots showed a clear distinction of the open chromatin landscapes between SAT and OVAT among all samples clearly highlighting depot-specific chromatin accessibility (Fig. 1a). Pearson correlation analysis and heatmap clustering of ATAC-seq samples demonstrated strong reproducibility among biological replicates from the same adipose tissue depot, highlighting data robustness (Supplementary Fig. S1a). We generated ⁓41–108 thousand unique filtered peaks in 16 adipose tissue samples from eight individuals (Supplementary Table S1). We identified 166.129 consensus peaks (accessible regions) by taking union of the peaks across all the samples and retaining those, which are appearing in more than one sample from eight individuals (Supplementary Table S1, Methods). Next, we examined the genomic distribution of the peaks among all the samples. The majority of peaks mapped to the following three main genomic regions: promoters, intronic and intergenic regions. Overall, genomic annotation of the peaks across all samples showed similar distribution further confirming the robustness of data (Fig. 1b). To compare our data with existing human adipose ATAC-seq datasets from ENCODE^71^ (subcutaneous origin) and Canon ME et al. (human subcutaneous adipose tissue, preadipocytes and adipocytes from the Simpson Golabi-Behmel Syndrome (SGBS) cell strain)^22^ we assessed peak overlaps. Adipose tissue peaks from ENCODE and Cannon ME et al. showed high overlap with our dataset (∼ 90%), whereas SGBS-derived preadipocytes and adipocytes displayed less than 50% overlap (Supplementary Fig. S1b). Fig. 1, panels c–d show IGV browser view of the ATAC-seq data from two representative, exemplary regions across all samples and adipose tissue depots.

Overall, we generated detailed maps of open chromatin from both adipose tissue depots and identified nuclear hotspots (peaks) with enriched chromatin accessibility in SAT and OVAT.

### Differential chromatin accessibility analysis reveals twice as many open chromatin regions in OVAT as compared to SAT

**Fig. 2:**
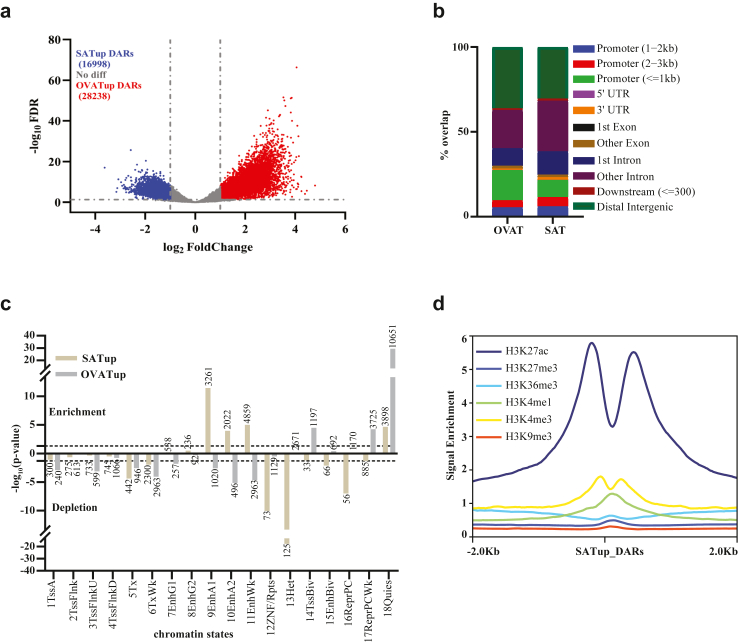
**Differentially Accessible Regions (DARs), overlap with roadmap adipose nuclei chromatin states.** a) Volcano plot of differential chromatin accessibility between SAT and OVAT depots. Points highlighted in red and blue show OVAT and SAT specific differential accessibility respectively (as defined by *log*_*2*_*(Fold Change)* > 1 and FDR adjusted *p-*value < 0.05). b) Genomic distribution of SAT and OVAT specific differentially accessible regions across all ATAC-seq mapped samples. c) Annotation and enrichment the differentially accessible regions between SAT(blue) and OVAT (red) with respect to roadmap adipose nuclei chromatin states (E063). Barplot represents -log_10_ enrichment or depletion *p*-values, and number of actual overlapping counts, for each state (Fisher’s exact test, two-sided). d) Histone marks enrichment (ChIP-seq signals from Roadmap subcutaneous Adipose tissue) around SATup DARs.

To test the hypothesis that our paired samples of SAT and OVAT confer potential depot-specific differentially open chromatin regions, we performed differential chromatin accessibility analyses. Collectively, we identified 45.236 differentially accessible regions (DARs) between SAT and OVAT (Supplementary Table S2). Among those, 28.238 regions were more accessible in OVAT (hereafter indicated as OVATup DARs) whilst 16.998 regions showed increased chromatin accessibility in SAT (hereafter indicated as SATup DARs) (Fig. 2a). Overall, we found about twice as many open chromatin regions in the visceral adipose tissue depot compared to the subcutaneous compartment. Further, when analysing the annotation of these regions according to the genomic distribution, we observed a higher proximal promoter (≦1 kb) enrichment for OVATup DARs (17.74%) as compared to SATup DARs (10.11%). Interestingly, we found accessible regions in SAT overrepresented in intronic regions (43.59%) compared to OVAT (32.84%). A substantial number of differential open chromatin regions were mapped into distal intergenic regions in both adipose tissue depots with SATup (30.49%) and OVATup (36.19%) DARs respectively (all data Fig. 2b; Supplementary Table S3).

### Chromatin states annotation analysis reveals an enrichment of SATup open regions in enhancer states

To assess the functional implications of differentially accessible regions (DARs), we performed an enrichment analysis against already annotated regulatory elements (chromatin states) in adipose tissue nuclei (E063) from the Roadmap Epigenomics 18-chromatin state model^53^ (Fig. 2c; Supplementary Table S4). These adipose nuclei chromatin states (E063) derived from female human subcutaneous abdominal adipose tissue aligns with our dataset, which also includes females and samples from both SAT and OVAT. Interestingly, we observed several statistically significant enrichments/depletions between SAT and OVAT: SATup DARs were significantly enriched in enhancer states, while OVATup DARs were significantly depleted (underrepresented) in these regions (9EnhA1, 10EnhA2, 11EnhWk; Fig. 2c). To further substantiate our enhancers findings, we employed histone enrichment analyses using ChIP-seq data for six core histone modifications (H3K4me3, H3K4me1, H3K36me3, H3K27ac, H3K27me3, and H3K9me3) from subcutaneous adipose tissue nuclei (Roadmap epigenomics).^53^ This analysis further strengthened our results, showing strong enrichment of the active enhancer mark H3K27 acetylation in SATup DARs (Fig. 2d). OVAT up DARs showed significant enrichment in bivalent transcription start site (14TSSBiv; Fig. 2c), Polycomb repressive (17ReprPCWk; Fig. 2c) and bivalent enhancers (15EnhBiv; not statistically significant) chromatin state, while SATup DARs were significantly underrepresented in bivalent enhancers (15EnhBiv; Fig. 2c) and Polycomb repressive states (16ReprPC, 17ReprPCWk; Fig. 2c). Bivalency is suggested to demarcate regulatory regions, which can undergo a rapid switch in the chromatin state upon cellular differentiation or tissue specific expression.^72^ Thus, these data might point towards bivalent regions playing a role in defining depot-specificity.

Both SAT and OVATup DARs showed similar trend of being underrepresented in transcription start sites (Tss) and transcribed regions (Tx). Additionally, a large number of DARs showed enrichment in quiescent chromatins state (18Quies with no histone marks; Fig. 2c) in both tissue depots with a much stronger enrichment in OVAT. Our findings are consistent with previous studies reporting an enrichment of accessible chromatin regions and differentially methylated regions (DMRs) in enhancer states from SAT derived roadmap adipose nuclei chromatin states.^22^^,^^73^ Nevertheless, it’s important to note that these overlaps rely on chromatin states from subcutaneous adipose tissue (E063), and for a comprehensive understanding of the overall visceral adipose tissue, we require chromatin states derived specifically from abdominal adipose tissue of visceral origin.

### Motif analysis in differentially accessible regions identifies CTCF as the most enriched motif in SAT

**Fig. 3:**
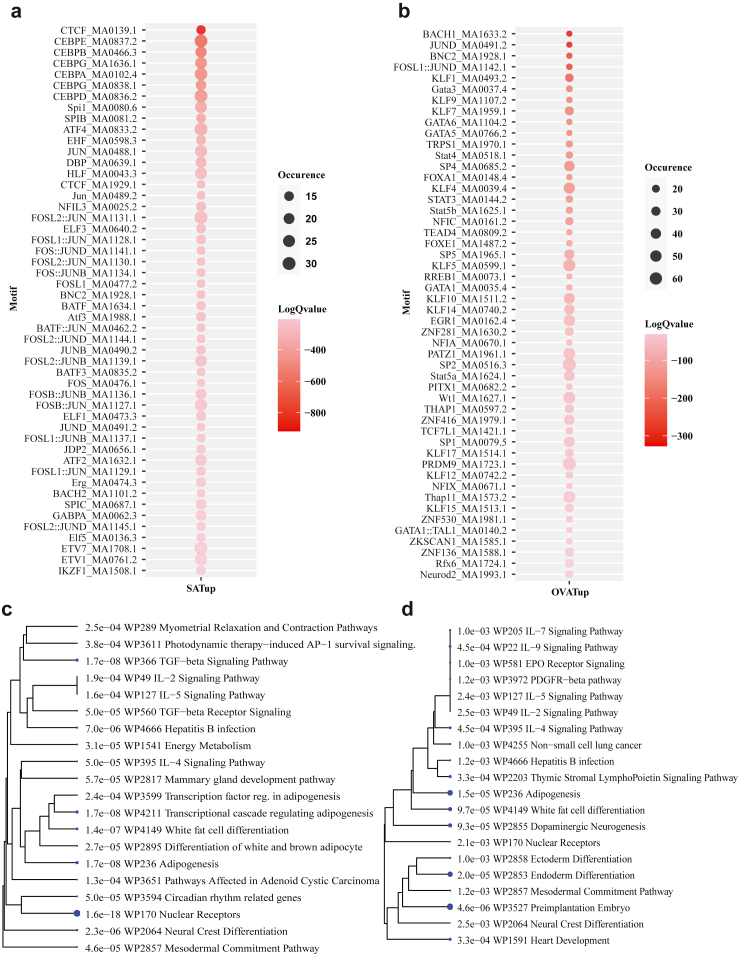
**Motif enrichment analysis in differentially accessible regions.** a and b) Dotplot of the significantly overrepresented motifs in DARs between SAT (left) and OVAT(right). The size of the circle indicates the percentage of DARs containing the motif, colour indicates the log Q-value. c and d) Hierarchical clustering trees of WikiPathways enrichment of c) SAT and d) OVAT specific transcription factors motifs. Hierarchical clustering tree summarizes the correlation among significant pathways listed in the Enrichment tab. Pathways with many shared genes are clustered together. Bigger dots indicate more significant *p*-values. Abbreviations: SAT = subcutaneous adipose tissue, OVAT = omental visceral adipose tissue.

Open chromatin regions enable binding of transcription factors (TFs) regulating the gene expression. To this end, we performed enrichment analyses for transcription factor motifs in DARs from SAT and OVAT. We identified 262 and 116 significantly enriched TF motifs (E-value ≤ 10 and TP% > 10%) in SAT and OVAT, respectively (Supplementary Table S5). In Fig. 3, panels a–b show the top 50 most significantly overrepresented TF motifs in differentially open regions of each adipose tissue depot (occurrence >10%). Overlap analysis of the identified motifs showed 226 and 80 TF motifs enriched in SAT and OVAT-specific accessible regions, respectively (Supplementary Table S6). Notably, the *CTCF* binding motif was most significantly overrepresented in SAT-specific regions (q-value 2.82e-399) present in about 14% of all SAT DARs (Fig. 3a). While *EMX2* had the highest occurrence, found in around 73% of SAT-specific accessible regions (q-value 0.00785). *EMX2* along with *ATF3, DMRT3,* and *ESR2* also showed upregulated expression in SAT supporting our previous findings.^8^ *EMX2* expression was also successfully validated in three independent validation cohorts (Supplementary Fig. S2 panels a–c). Further, among the top significant TF motifs in SAT, we found enrichment for CEBP family members (such as *CEBPA, CEBPB,* and *CEBPD*), AP1 TFs (including ATF, JUN and FOS subfamilies) and various ETS family members (including *EHF, ELF1, ELF3, ETV1, ETV6, ETV7, GABPA,* and *SPIC etc.*), which have previously been associated with adipose tissue development.^11^^,^^74^, ^75^, ^76^ Pathway analysis of transcription factors corresponding to the 226 motifs of SAT-specific accessible regions revealed an enrichment for transcriptional cascades regulating TGF-beta signalling pathway, nuclear receptors, adipogenesis, energy metabolism, and circadian rhythm related genes (Fig. 3c, Supplementary Table S7). Conversely, in OVAT-specific accessible regions (116 motifs), we found enrichment for binding sites of the GATA family (*GATA1*, *GATA3, GATA5, GATA6*), SP/KLF TF family (e.g., *KLF4, KLF5, KLF14, SP1,* and *SP2*), STAT family (*STAT3, STAT4, STAT5A,* and *STAT5B*), *TEAD4, PITX,* and *WT1* etc. *BACH1* motif showed the most significant enrichment (q-value = 6.4E-143) with ∼10% occupancy, while *NFATC3* had the highest occupancy at ∼73% of OVAT-specific accessible regions (q-value = 0.000433; Fig. 3b). Additionally, seven transcription factors (*BNC2, GATA5, GATA6, KLF5, PITX1, SOX6,* and *WT1*) corresponding to OVAT enriched motifs (116) were also upregulated in OVAT and several were successfully validated in three independent validation cohorts (Supplementary Fig. S3 panels a–c). Pathway analysis linked OVAT-enriched motifs to adipogenesis, white fat cell differentiation, endoderm differentiation, heart development, and *IL9* signalling etc (Fig. 3d; Supplementary Table S7).

### Gene expression analysis corroborates previous data on differential expression between SAT and OVAT

To explore the functional implications of differential chromatin accessibility on gene expression, we performed transcriptome-wide expression analysis in paired SAT and OVAT samples from five of the same individuals. Differential expression analysis identified 1.368 differentially expressed genes (DEGs) (log_2_(Fold Change) > 1 and FDR adjusted *p-*value < 0.05; Supplementary Fig. S4a), with 923 genes upregulated in OVAT (hereafter indicated as OVATup DEGs) and 445 in SAT (hereafter indicated as SATup DEGs; Supplementary Fig. S4a and Table S8). These findings largely align with previously published studies from our group and others.^8^^,^^77^, ^78^, ^79^, ^80^, ^81^, ^82^, ^83^ In SATup DEGs, we found developmental, and obesity linked genes such as the well described HOXC cluster (*HOXC4, HOXC5, HOXC6, HOXC8, HOX9,* and *HOXC10*), TBX family (*TBX5, TBX15,* and *TBX18*), and IRX family (*IRX1, IRX2, IRX3,* and *IRX5*), highlighting their important role in adipose tissue biology and obesity pathogenesis. OVATup DEGs included the kallikrein-related peptidases (KLKs) family (*KLK5, KLK7,KLK10,* and *KLK11*), the Keratin (KRT) family (*KRT5, KRT7, KRT8, KRT18,* and *KRT19*), and several other obesity associated genes that were previously reported in other studies (including *ALOX15, UPK1B, GATA5, LRRN4, ANXA8, ANXA8L1, ISL1, MUC16, CKMT1A, CKMT1B, CDH3, CDH1,* and *CLDN1*).^8^^,^^81^^,^^84^, ^85^, ^86^, ^87^, ^88^, ^89^, ^90^ Importantly, OVAT showed approximately twice as many upregulated genes compared to SAT, consistent with our results from ATAC-seq data. Given the heterogeneity of adipose tissue cell types, we refined our findings by comparing them with in-house data on differentially expressed genes (DEGs) from purified adipocytes of subcutaneous and visceral origin in five subjects. This analysis identified 403 DEGs between SAT and OVAT that were also found differentially expressed in the corresponding purified adipocytes (Supplementary Fig. S4b). Downstream enrichment analysis using various gene set libraries from enrichr^61^ linked these adipocyte-adipose tissue common DEGs to obesity, blood pressure, cardiovascular diseases (enrichr; PhenGenl Association2021) (Supplementary Fig. S4c) and an enrichment of Adipocyte as a cell type (enrichr; Human Gene Atlas) (Supplementary Fig. S4d).

### Integrated analysis of open chromatin regions and transcriptome

**Fig. 4:**
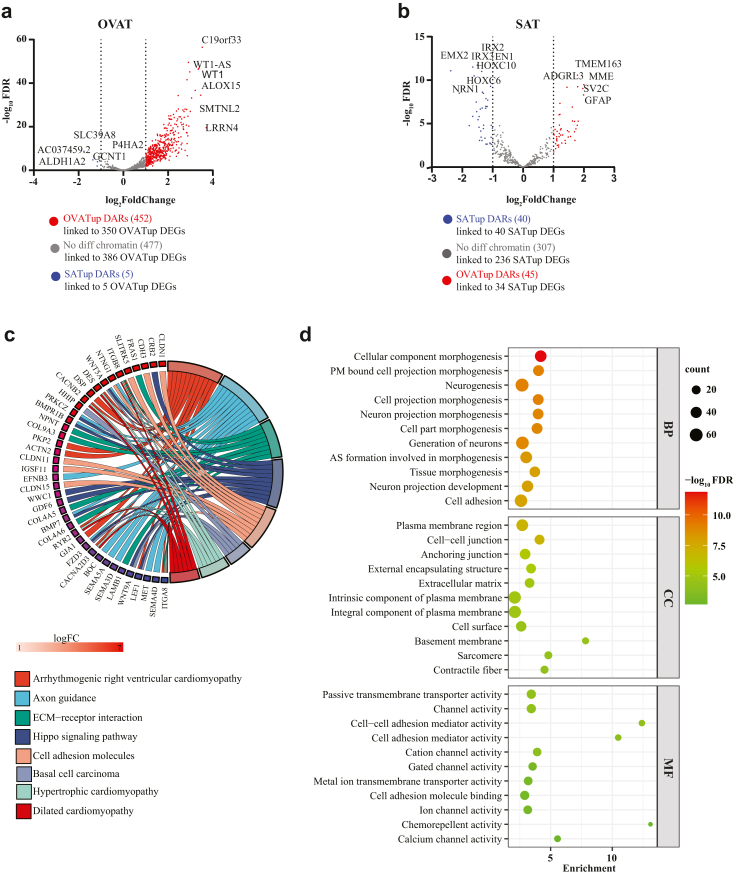
**Integration of the ATAC-seq and RNA seq data for promoter analysis.** a) Volcano plot of all genes with higher gene expression in OVAT and overlapping with differentially accessible regions in OVAT (red) versus SAT (blue) or regions without differential accessibility (grey) in their promoter region (defined as ±1 kb of their TSS). b) Volcano plot of all the genes with higher gene expression in SAT and overlapping with differentially accessible regions in SAT (blue) versus OVAT (RED) or regions without differential accessibility (grey) in their promoter region (defined as ±1 kb of their TSS). c) Circos plot of the KEGG pathway analysis for the OVATup DEGs having an OVATup DAR in their promoter region (from panel a (red) of the Fig. 4). d) Bubble plot showing the top Gene Ontology (GO) terms biological processes (BP), cellular component (CC) and molecular function (MF) significantly enriched in OVATup DEGs having an OVATup DAR in their promoter region (from panel a (red) of the Fig. 4). Size of the bubble represents the gene count whereas the colour represents the -log_10_FDR. Abbreviations: OVATup DAR = differentially accessible regions more open in omental visceral adipose tissue, OVATup DEG = differentially expressed genes upregulated in omental visceral adipose tissue; SATup DAR = differentially accessible regions more open in subcutaneous adipose tissue; SATup DEG = differentially expressed genes upregulated in subcutaneous adipose tissue.

To investigate the relationship between depot-specific gene expression and chromatin accessibility, we integrated our RNA-seq and ATAC-seq data focussing on the proximal promoter regions (±1000 bp of TSS) of the expressed genes. This analysis resulted in several subgroups, depending on gene expression and differential chromatin accessibility status of all regions associated with the gene promoter (Supplementary Table S9; Methods). In line with our findings from genomic annotation of DARs, we found a high overlap between open chromatin regions in OVAT with genes upregulated in OVAT reflecting the enrichment in promoter regions. Among the 923 OVAT-upregulated genes, 350 were associated with 452 OVAT-specific open chromatin regions (Fig. 4a, Supplementary Table S10). Additionally, 386 OVAT-upregulated genes were linked to 477 chromatin regions that are similarly accessible in both adipose tissue depots (Fig. 4a). In contrast, the overlap was less pronounced in SAT. For the total of 445 SAT-upregulated genes, only 40 overlapped with 40 SAT-specific open chromatin regions, while 236 were lined to 307 accessible regions in both depots (Fig. 4b, Supplementary Table S10). Interestingly, 930 non-differentially expressed genes were linked to 1023 OVAT-specific open chromatin regions while 280 such genes corresponded to 288 SAT-specific open chromatin regions (Supplementary Fig. S5). These findings highlight depot-specific differences in the relationship between promoter accessibility and gene expression.

### Pathway enrichment and GO-term analyses

To explore the functional implications of our findings, we performed pathway enrichment and gene ontology (GO) analyses. Given the limited overlap of DEGs with SATup DARs open chromatin regions (N = 40), we focused on our findings from OVAT (N = 350). KEGG pathway analysis revealed enrichment in pathways associated with cardiomyopathies including arrhythmogenic right ventricular, hypertrophic, and dilated cardiomyopathy driven by genes such as *ITGA8, ITGB8, DES, DSP, CACNB2, CACNA2D3, RYR2* and *ACTN2*. Additionally, pathways enriched in OVAT included hippo signalling, extracellular matrix (ECM) receptor interaction, cell adhesion molecules, and axon guidance (Fig. 4c; Supplementary Table S11). Gene Ontology (GO) analysis further supported these findings, showing enrichment for terms related to extracellular matrix, neurogenesis, and cell adhesion (Fig. 4d; Supplementary Tables S12–S14). To further substantiate these findings, we set out to validate genes originating from these pathways (such as *COL4A5, COL4A6, LAMB1, NPNT, BMP7, PRKCZ, WNT5A, SEMA5A, SEMA3D*, and *SEMA4D*), which exhibited increased chromatin accessibility and expression in OVAT. We successfully validated the direction of gene expression in adipose tissue for several of these targets in an independent large cross-sectional validation cohort (Supplementary Fig. S6).

### Super enhancers associated with open chromatin in SAT control ECM organization and pattern specification

**Fig. 5:**
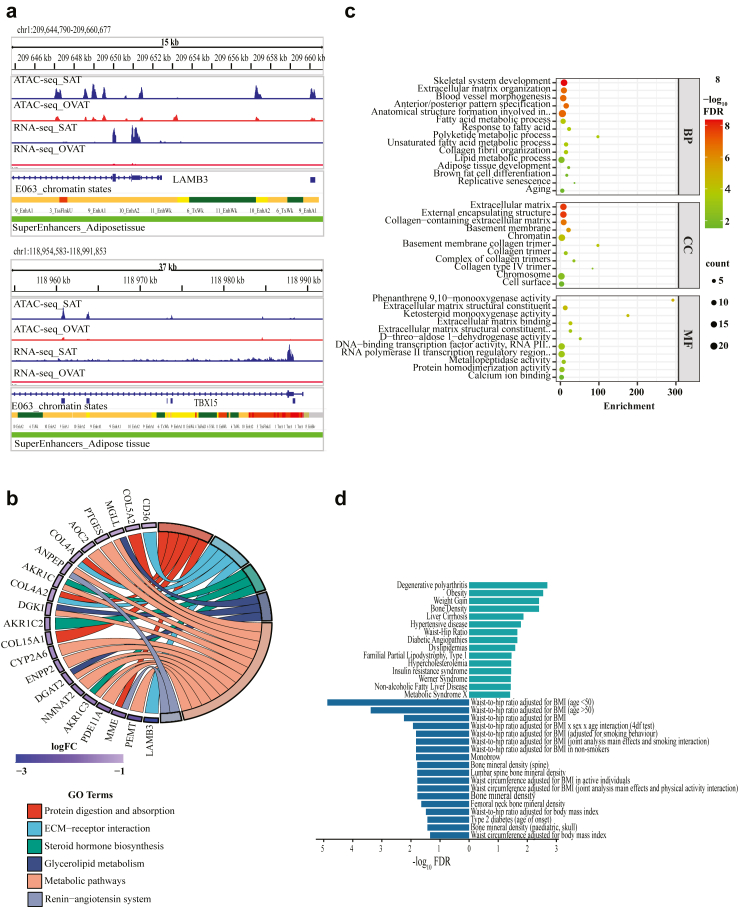
**Super Enhancer Analysis; Overlap of regions more open in SAT with adipose tissue Super Enhancers (SEs).** a) IGV browser view of the two representative regions harbouring LAMB3 and TBX15 genes and having a chromatin region more open in SAT overlapping with an adipose nuclei super enhancer with differentially expressed gene in close vicinity. b) Circos plot of the KEGG pathway analysis for upregulated genes in SAT that are associated with more open chromatin regions and overlap with a super enhancer. c) Bubble plot showing the most relevant Gene Ontology (GO) terms biological processes (BP), cellular component (CC) and molecular function (MF) significantly enriched among upregulated genes in SAT that are associated with more open chromatin regions and overlap with a super enhancer (97). Size of the bubble represents the gene count whereas the colour represents the -log_10_FDR. d) Barplot showing Enricher based analysis of genes upregulated in SAT in regions that are more open in SAT and fall under the control of a super enhancer (97). Right bars panel shows selected enriched terms (for the input gene set “N = 97”) from Disease Gene association (DisGeNET) gene set library. Left bars panel shows top enriched terms (for the input gene set “N = 97”) from GWAS catalogue gene set library which contains variants associated with different human diseases traits. The enriched terms are displayed as -log_10_FDR values.

Our genomic annotation and chromatin state analysis of differentially accessible regions (DARs) revealed a high enrichment for enhancers in SAT. To further characterize these SAT-specific DARs, we examined their overlap with pre-defined super enhancers using adipose tissue data from the super enhancer database SEdb.^60^ We identified 2.907 SATup DARs overlapping with 3.650 super enhancers defined in adipose tissue (hereafter indicated as SE-linked SATup DARs; Supplementary Table S15). Notably, ∼48% of these super enhancers linked DARs (1384/2907) were annotated to intronic regions (Supplementary Table S15). Intronic enhancers are suggested to play critical roles in defining cellular identity.^91^ Fig. 5a shows two representative loci from *LAMB3* and *TBX15* genes, that are upregulated in SAT, overlapped with an adipose tissue super enhancer.

We annotated 3797 genes (defined by SEdb and our data) linked to these super enhancers in SAT and identified 97 genes among them that overlapped with SAT-upregulated genes, suggesting depot-specific functional relevance (Supplementary Table S15). KEGG Pathway analyses of these genes highlighted biologically relevant terms such as protein digestion and absorption, extracellular matrix organization, metabolic pathways, glycerol-lipid metabolism, steroid hormone biosynthesis with the following set of genes *AKR1C1, AKR1C2, AKR1C3, ANPEP, AOC2, CD36, COL15A1, COL4A1, COL4A2, COL5A2, CYP2A6, DGAT2, DGKI, ENPP2, LAMB3, MGLL, MME, NMNAT2, PDE11A, PEMT,* and *PTGES* (Fig. 5b; detailed lists in Supplementary Table S16). GO-term analyses further emphasized the roles of these super enhancers linked genes in ECM organization, skeletal system and pattern organization, blood vessel morphogenesis, fatty acid metabolic process, adipose tissue development, replicative senescence, and ageing (Fig. 5c depicting most relevant GO terms; detailed lists in Supplementary Tables S17–S19). Enrichr^61^ analysis using DISGeNET and GWAS catalogues reinforced disease and GWAS associations (Fig. 5d). Relevant enriched terms in DISGeNET included obesity, insulin resistance, dyslipidaemia and non-alcoholic fatty liver disease, whilst GWAS enrichment analysis returned important associations with anthropometric traits such as body mass index (BMI)-adjusted waist-to-hip ratio (WHR), waist circumference (WC) and type 2 diabetes (T2D) (Fig. 5d; Supplementary Tables S20 and S21). These findings highlight the functional and clinical relevance of depot-specific super enhancer-linked genes.

### Correlation of gene expression with clinical variables

Our findings so far highlighted distinct chromatin accessibility patterns and their impact on depot specific gene expression in two human adipose tissue depots. We identified two key sets: one comprising 350 OVAT-upregulated genes linked to more accessible chromatin at promoters in OVAT and another with 97 SAT-upregulated genes associated with super enhancers overlapping with open chromatin regions in SAT.

**Fig. 6:**
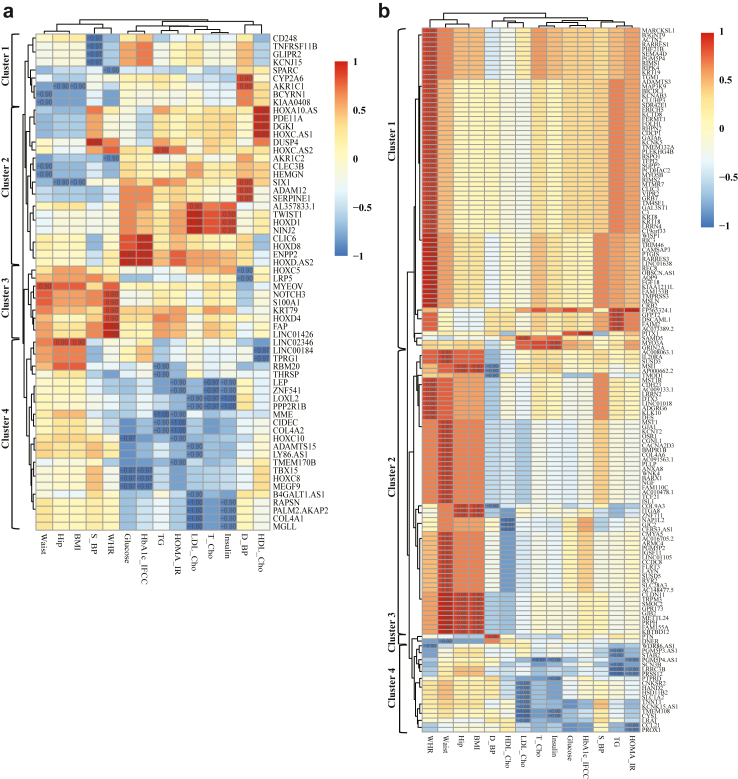
**Correlation analysis of gene expression with clinical variables.** a) Heatmap of the correlation of expression values (TPMs) in SAT with clinical variables. Correlation coefficient (r) values are indicated for the significant correlations and colour represents positive or negative correlation; significance level cutoff is set at *p*-value < 0.05. Genes included in this analysis are the ones upregulated in SAT, in regions more accessible in SAT and under control of a super enhancer) (N = 97) Abbreviation: TPM = transcript per million. b) Heatmap of the correlation of expression values (TPMs) of upregulated genes in OVAT with clinical variables. Correlation coefficient (r) values are indicated for the significant correlations and colour represents positive or negative correlation; significance level cutoff is set at *p*-value < 0.05. Genes included in this analysis are the ones upregulated in OVAT, and their promoter regions overlap with chromatin regions being more open in OVAT (N = 350) (promoter region defined as ±1 kb of their TSS) Abbreviation: TPM = transcript per million.

To explore clinical relevance, we correlated the expression of these genes with obesity-related clinical variables from the same set of patients (N = 5). Genes with significant clinical correlation were visualized in a heatmap and resolved into four distinct clusters (Fig. 6 panels a–b). Among the 97 SAT-upregulated genes, 62 genes (∼64%) showed significant correlations with clinical variables such as fasting glucose, insulin, HOMA-IR, LDL cholesterol, HDL cholesterol, and triglycerides (Fig. 6a). These genes included *AKR1C1*, *AKR1C2*, *COL4A1*, *COL4A2*, *CYP2A6*, *DGKI, ENPP2*, *MGLL*, *MME* and *PDE11A,* many of which were also enriched in our KEGG pathways related to metabolic functions (Fig. 5b). Several of these targets related to these pathways in Fig. 5b (such as *AKR1C1, AKR1C3, ANPEP, AOC2, CYP2A6, DGAT2, DGKI, ENPP2, MGLL, NMNAT2, PDE11A, PEMT*, and *PTGES*) were taken forward to validation analyses in a large cross-sectional independent cohort. Upregulated gene expression in SAT along with correlation to clinical variables were largely validated in these analyses (Supplementary Fig. S7 and Table S22). Genes in cluster 4 demonstrated a negative correlation with metabolic measures such as fasting plasma glucose, fasting plasma insulin, and HOMA-IR as well as variables associated with lipid metabolism such as low-density lipoprotein cholesterol (LDL-c), high density lipoprotein cholesterol (HDL-c) and triglycerides (TG): (*ADAMTS15*, *B4GALT1.AS1*, *LINC00184*, *LY86.AS1*, *RBM20*, *THRSP*, *TMEM170B*, *TPRG1*, *CIDEC*, *COL4A1*, *COL4A2*, *HOXC10*, *HOXC8*, *MEGF9*, *MGLL*, *MME*, *PALM2.AKAP2*, *RAPSN*, *TBX15*, *LEP*, *LOXL2*, *PPP2R1B*, and *ZNF541*) (Fig. 6a). While cluster 1 and 2 including genes *AKR1C1, AKR1C2, BCYRN1, CLEC3B, HEMGN, KIAA0408, SIX1 and SPARC* showed a negative correlation to anthropometric variables such as waist circumference, hip circumference and WHR. Conversely, cluster 3 contained genes like *MYEOV, NOTCH3, S100A1, KRT79, HOXD4, FAP and LINC01426,* showing a positive correlation with anthropometric variables such as waist and WHR. These data also corroborate previous studies; as most of these genes were previously linked to adipose tissue biology and obesity.^80^^,^^92^, ^93^, ^94^, ^95^, ^96^, ^97^, ^98^, ^99^, ^100^ Interestingly, when performing similar correlation analyses for the 350 OVAT-upregulated genes, a substantial subset of genes positively correlated with fat distribution measures such as waist circumference, hip circumference, and WHR (cluster1 and 2). For example, 69 out of 350 genes showed a positive correlation with WHR. Notable genes included those involved in metabolic pathways (*GAL3ST1*, *ADAMTS3*, *FOLH1, PTGIS, SGPP2, MTMR*), extracellular lipid organization (*TGM1, KRT19, KRT18, KRT8*), metabolic diseases (*VIPR2, PTGIS, LRRN2, AQP9, GATA6, MTMR7, PHF21B, RIMS2, TGM1, C3, KCTD8, RIMS1, ADAMTS3, FOLH1, MYO5B, FGF18, ADGRG6, CDH23*) and type 2 diabetes (*C3, RIC3, B3GNT9, DES, FOLH1, PTGIS, GATA6, MST1R*) *etc.* Several genes which showed enrichment in our promoter KEGG pathway analysis (such as *ACTN2*, *BMPR1B*, *CACNA2D3*, *CLDN11*, *COL4A6*, *COL9A3*, *CRB2*, *DES*, *GJA1*, *IGSF11*, *ITGA8*, *RYR2*, *SEMA4D*) (Fig. 4c) were present in these clusters, predominantly cluster 2 (Fig. 6b). Additionally, a set of nine genes in cluster 4 including *TNNT1, HSD11B2, SLC1A2, HAND2, CNKSR2, KCNK15-AS1, LRAT, TMEM108* and *CYS1* showed a negative correlation with LDL cholesterol (Fig. 6b). This set includes genes that are involved in lipid response and were previously linked to obesity such as *HAND2, HSD11B2, TNNT1, LRAT,* and *TMEM108*.^8^^,^^101^^,^^102^ Collectively, our data shows that depot specific expressed genes either regulated by differentially accessible super enhancers in SAT or promoters in OVAT, correlate with clinical variables important for anthropometric variables as well as to insulin, glucose, and lipid metabolism.

## Discussion

Our work provides detailed genome-wide chromatin accessibility profiles along with the transcriptomic landscape in intra-individually paired human samples of OVAT and SAT from individuals with obesity. Distinct chromatin architecture at *cis*-regulatory regions is a key factor in driving tissue-specific expression and maintaining cellular identity and function.^103^ In this study, we observed that chromatin accessibility is clearly adipose tissue depot-specific. The visceral adipose tissue compartment shows many more relaxed, accessible chromatin regions compared to its subcutaneous counterpart with almost twice as many open chromatin regions than in SAT. This is in line with around two-fold higher number of genes being higher expressed in the visceral depot. By integrating chromatin openness with gene expression levels, we found that genes upregulated in SAT were mostly related to adipose tissue biology and metabolic pathways, whilst genes higher expressed in OVAT are associated with neurogenesis, cardiomyopathies, ion channel activity and cell adhesion molecules.

Transcription factors regulate gene expression by binding to regulatory regions, influencing key biological processes, including cell identity and tissue development.^104^ In this study, our motif analysis identified specific TF motifs enriched in the differentially accessible chromatin regions of SAT and OVAT that might play pivotal roles in modulating adipose tissue biology. For example, the most significant enrichment of *CTCF* in SAT aligns with recent reports linking *CTCF* to transgenerational epigenetic inheritance of obesity and its potential role in shaping adipose depot characteristics, such as pear-shaped fat distribution in women, which is metabolically favourable.^105^^,^^106^ Similarly, *EMX2*, which exhibited the highest motif occupancy (73%) and showed upregulated expression in SAT, was previously identified by us as hypermethylated in OVAT and upregulated in SAT, further reinforcing its potential as a depot-specific regulator.^8^ Moreover, we observed enriched motifs for other TFs with known roles in adipose biology, such as the circadian regulators (e.g., *RORA*, *ATF4*, and *PPARG*) in SAT, which have been associated with adipocyte differentiation and adipose tissue function.^107^, ^108^, ^109^

In OVAT, *BACH1* and *NFATC3* were among the most significantly enriched and highest occurrence motifs respectively, highlighting their possible contributions to visceral adiposity and its associated inflammation. *NFATC3* has established roles in macrophage polarization and immune cell activity, processes critical to obesity-induced inflammation.^110^^,^^111^ *BACH1*, has been linked to ferroptotic FGF21 secretion, a mechanism shown to mitigate obesity-related traits in mice.^112^ Moreover, in mice, *BACH1* is shown to be involved in regulating oxidative stress,^113^ a factor in obesity-associated insulin resistance, while its hepatic deletion improves insulin signalling and glucose metabolism by modulating key protein interactions.^114^ This further underscores the relevance of *BACH1* TF is the context of obesity. Notably, *KLF14,* despite its low expression (average TPM ∼1), occupied 40% of OVAT-specific open regions in our cohort (female), aligning with reports linking its genetic variability and limited regulatory capacity to female body shape and metabolic health.^115^^,^^116^ Additionally, among OVAT-enriched motifs, we observed upregulated expression of TFs such as *GATA5*, *GATA6*, *KLF5*, and *WT1*, consistent with their known roles in visceral fat development and adipocyte differentiation.^117^, ^118^, ^119^, ^120^ Importantly, effect directions of upregulated TFs such as *EMX2* (in SAT) and *GATA5, GATA6*, *KLF5*, and *WT1* (in OVAT) were validated in three validation cohorts confirming and underlining their role in obesity and metabolic dysfunction. Collectively, our findings not only emphasize the depot-specific transcriptional landscapes and mechanistic pathways for further exploration but also highlight candidate TFs for therapeutic targeting in the context of obesity and metabolic health.

Super enhancers are a unique class of enhancer clusters with well-studied roles in defining cellular identity and are implicated in disease pathogenesis, including cancer.^121^ In our study, chromatin state analysis showed significant enrichment for enhancer elements, supported by high levels of H3K27 acetylation, an active enhancer mark (Fig. 2d). Notably, we found that numerous open chromatin regions in subcutaneous adipose tissue (SAT) align with super enhancers specific to adipose tissue, revealing roughly 3000 regions linked to 97 upregulated genes enriched in metabolic processes and extracellular matrix organization. Our data suggests that such organization seems to be under the control of super enhancers in SAT. In contrast, the loss of extracellular matrix regulation and other features governed by super enhancers in SAT might contribute to the dysfunction observed in OVAT. These findings align with previous studies linking extracellular matrix dysregulation to metabolic diseases including obesity.^122^^,^^123^ Additionally, genes associated with these SAT super enhancers, such as *PEMT, PDE11A, AKR1C3, NMNAT2, DGAT2, ENPP2 CYP2A, DGKI, AKR1C1, ANPEP, AOC2, PTGES* and *MGLL* are known for their roles in metabolic health; and dysregulation has been associated with obesity and insulin resistance.^124^, ^125^, ^126^, ^127^, ^128^, ^129^, ^130^, ^131^, ^132^ Along those lines, in our study downregulation of these genes in OVAT might point towards metabolic impairment and complications.

Interestingly, we observed enrichment of bivalent chromatin states in both TSS (significant enrichment) and enhancer regions (enrichment but not statistically significant), in open chromatin regions more accessible in OVAT. This bivalency, characterized by both activating and repressive histone marks,^133^ suggests a “poised” chromatin state that, while primed in SAT, may remain inactive but accessible in OVAT, potentially indicating metabolic vulnerabilities.

Our integrated chromatin accessibility and transcriptome analysis revealed upregulated genes in OVAT, enriched in pathways related to cardiomyopathies, axon guidance, extracellular matrix (ECM) organization, Hippo signalling, and cell adhesion pathways critical to adipose tissue function and linked to obesity and metabolic disorders.^134^, ^135^, ^136^, ^137^ The hippo signalling pathway and its ECM crosstalk is suggested to play an important role in aberrant adipose tissue homoeostasis upon caloric excess,^137^ potentially contributing to OVAT fibrosis and metabolic imbalance. Several candidate genes from these pathways (Fig. 4c) such as collagen IV (*COL4A5* and *COL4A6*), laminins (*LAMB1*), *NPNT*, *BMP7*, *PRKCZ*, and *WNT5A* have been implicated in adipose tissue remodelling, inflammation and other obesity linked traits.^138^, ^139^, ^140^ Genetic impairments of axon guidance molecules have been implicated in early onset obesity.^141^^,^^142^ We also found several axon guidance genes, particularly semaphorin family members (*SEMA5A*, *SEMA3D*, *SEMA4D*), upregulated in OVAT with increased chromatin accessibility. These genes, involved in leptin-related neuronal pathways, are linked to obesity and metabolic disease,^141^^,^^143^ suggesting a depot-specific effect on neuronal outgrowth in OVAT. Our data were further substantiated by validating a higher gene expression in OVAT for several of these genes in an independent validation cohort (Supplementary Fig. S6). Additionally, we also identified an interesting group of non-differentially expressed genes in both OVAT (N = 930) and SAT (N = 280) with accessible promoter regions, possibly representing open but inactive chromatin regions that may require further epigenetic activation (Supplementary Fig. S5).

Our findings reveal distinct depot-specific chromatin accessibility patterns shaping gene expression in human adipose tissues, with clinical relevance to obesity-related traits. These results emphasize the distinct biological roles of depot-specific gene regulation and highlight a subset of genes with potential as biomarkers or therapeutic targets for metabolic disorders, offering avenues for follow up studies to address obesity and its complications.

Although this study describes an extensive genome wide chromatin accessibility mapping in paired human SAT and OVAT samples, it is limited at several aspects. We have analysed whole adipose tissue biopsies, which are highly heterogeneous and contain multiple cell types including immune, vascular, stem and progenitor cells. Consequently, some of the observed effects might stem from non-adipocyte cell types but will nevertheless be relevant for understanding the development of obesity and its related metabolic complications. Of note, we have included purified adipocytes in our RNA-seq analyses, largely corroborating our findings. All individuals are of Caucasian ethnicity and the results of this study may not be generalized to other ethnicities. Additionally, since we included only individuals with obesity and no normal-weight controls, it is limiting our interpretation on whether the observed effects are unique to obesity or reflect general depot-specific characteristics.

In conclusion, our study provides detailed chromatin accessibility maps along with transcriptome profiles enabling a direct comparison of intra-individually paired samples of SAT and OVAT. Moreover, we provide a rich compendium of differentially open *cis*-regulatory regions and annotated genes, representing an extensive resource for further studies of genes residing in differentially open chromatin regions and being potentially involved in the pathogenesis of obesity, fat distribution, and related metabolic comorbidities.

## Contributors

YB conceived and designed the study and received funding. SS provided crucial input on and designed the entire analysis strategy, SS contributed with important discussions. LlCP performed the entire laboratory experiments related to ATAC-seq, contributed to biobanking, first steps of analysis and important discussions. SS and TV performed the bioinformatic analysis in the discovery cohort. TR generated RNA-seq data from fat tissues in the discovery cohort and contributed to important discussions. JW provided preliminary bioinformatic support and was involved in data analysis discussions. SS, TV and JW have verified the underlying data. MBD provided technical assistance with respect to fat tissue biobank. AC participated in statistical analysis related to chromatin states. TM, JAK, MS, JH and JKH provided fat tissue biopsies. MB and CW contributed human data from LOBB validation cohort and AH and AG analysed the data bioinformatically. TGV provided technical assistance with clinical data interpretations and coordinated the availability of fat tissue biopsies. SS wrote the manuscript draft. YB wrote the final manuscript. All authors contributed to the final version by proof reading and editing of the manuscript and approved the manuscript.

## Data sharing statement

The dataset supporting the final results and major findings of the study is included in the main article as well as Supplementary files. Moreover, controlled access could be provided to sequencing data of the discovery cohort upon request via TSD (Services for Sensitive Data), which is designed for storing and post-processing sensitive data in compliance with the Norwegian “Personal Data Act” and “Health Research Act”. The human RNA sequencing data from the LOBB have not been deposited in a public repository due to restriction by patient consent but are available from MB on request.

## Declaration of interests

MB received personal honoraria from Amgen, AstraZeneca, Bayer, Boehringer-Ingelheim, Lilly, Novo Nordisk, Novartis, and Sanofi as well as payments from Boehringer-Ingelheim to the institution. The other authors on the manuscript declared no competing interests.

## Appendix ASupplementary data

Supplementary Tables S1–S22 **Table S1:** ATAC-seq sequencing metrices of human paired samples of subcutaneous (SAT) and omental visceral (OVAT) adipose tissue. **Table S2:** List of the Differentially Accessible Regions (DARs) between two fat depots called as SATup DARs and OVATup DARs. **Table S3:** Genomic distribution of the differentially accessible regions between SAT and OVAT. **Table S4:** Percentage overlap of the Differentially Accessible Regions (DARs) with roadmap epigenomics Adipose nuclei chromatin states. **Table S5:** Lists of overrepresented Transcription Factors (TFs) motifs in DARs between two fat depots using SEA. Motif enrichment analysis was performed against JASPAR2022_CORE_vertebrates_non-redundant database using MEME-suit Simple Enrichment Analysis of motifs (SEA) Version 5.5.1 with default settings (Expected value (E) ≤ 10 (41). The enrichment E-value of a motif is its adjusted *p*-value multiplied by the number of motifs in the input. TP% = The percentage of primary sequences matching the motif with scores greater than or equal to the optimal match score threshold. Qvalue = The *q*-value is the minimum False Discovery Rate (FDR) required to consider this motif significant. **Table S6:** Lists of overrepresented Transcription Factors (TFs) motifs in DARs which are specific to SAT (226), OVAT (80) and common between two depots (36). **Table S7:** List of enriched Wikipathways in overrepresented Transcription Factors (TFs) motifs which are specific to SAT (226), and OVAT (80). **Table S8:** List of the Differentially Expressed Genes (DEGs) between two fat depots called as SATup DEGs and OVATup DEGs. **Table S9****:** ATAC-seq and RNA-seq integrated analysis grouping by taking all the expressed genes in SAT and OVAT and chromatin accessibility in their proximal promoters. **Table S10:** List of the Genes specifically up regulated in OVAT (N = 350) and SAT (N = 40) with a more open chromatin region in their promoters. **Table S11:** KEGG pathway enrichment analysis for the OVATup DEGs linked to OVATup DARs through integrated ATAC-seq RNA-seq analysis (promoter analysis). **Table S12:** Gene Ontology (GO) terms analysis: Biological pathways (BP) enriched for the OVATup DEGs linked to VATup DARs through integrated ATAC-seq RNA-seq analysis (promoter analysis). **Table S13:** Gene Ontology (GO) terms analysis: Cellular Component (CC) enriched for the VATup DEGs linked to OVATup DARs through integrated ATAC-seq RNA-seq analysis (promoter analysis). **Table S14:** Gene Ontology (GO) terms analysis: Molecular Function (MF) enriched for the OVATup DEGs linked to VATup DARs through integrated ATAC-seq RNA-seq analysis (promoter analysis). **Table S15:** List of Adipose tissue Super Enhacers from SEdb 2.0 overlapping with SATup DARs along with their annotated genes. **Table S16:** KEGG pathway enrichment analysis for the SATup DEGs linked to Super Enhancers overlapping SATup DARs. **Table S17:** Gene Ontology (GO) terms analysis: Biological pathways (BP) enriched for the SATup DEGs linked to Super Enhancers overlapping SATup DARs. **Table S18:** Gene Ontology (GO) terms analysis: Cellular Component (CC) enriched for the SATup DEGs linked to Super Enhancers overlapping SATup DARs. **Table S19:** Gene Ontology (GO) terms analysis: Molecular Function (MF) enriched for the SATup DEGs linked to Super Enhancers overlapping SATup DARs. **Table S20:** Enrichr DISGeNET enrichment analysis for the SATup DEGs linked to Super Enhancers overlapping SATup DARs. **Table S21:** Enrichr GWAS catalogue 2019 enrichment analysis for the SATup DEGs linked to Super Enhancers overlapping SATup DARs.

Supplementary Table S22 **Table S22:** Clinical correlation of genes linked to metabolic pathways and upregulated in subcutaneous adipose tissue in a cross-sectional validation cohort. The human cross-sectional cohort comprises paired samples of OVAT and abdominal SAT from 1,480 individuals of the Leipzig Obesity Biobank (LOBB) including individuals with obesity (N = 1449) and without obesity (N = 31). Spearman correlation analyses were conducted to assess the relationship between gene expression values (log TMM, adjusted for age and sex) and clinical parameters in the cross-sectional validation cohort of omental visceral adipose tissue (OVAT). *p*-value legend: <0.001 (∗∗∗), <0.01 (∗∗), <0.05 (∗).

Supplementary Fig. S1 **Fig. S1: a) ATAC-seq Data analysis: Clustered heatmap of Pearson correlation coefficients for SAT and OVAT ATAC-seq data across all biological replicates.** Correlation is based on normalized *log* transformed read counts. **b)** Barplot showing percentage overlap of ATAC-seq peaks from different adipose tissue based datasets with our consensus peaks (166.129).

Supplementary Fig. S2 **Fig. S2: a) Validation of transcription factor gene expression upregulated in abdominal subcutaneous adipose tissue (SAT) in a cross-sectional validation cohort.** The human cross-sectional cohort comprises paired samples of OVAT and abdominal SAT from 1,480 individuals of the Leipzig Obesity Biobank (LOBB) including individuals with obesity (N = 1449) and without obesity (N = 31). Parametric hypothesis testing (Welch’s one way ANOVA; Games-Howell post-hoc Test) were used and the pairwise *p*-values were corrected for multiple inference using the Hommel’s methods. **b) Validation of transcription factor gene expression upregulated in abdominal subcutaneous adipose tissue (OVAT) in a bariatric surgery cohort before and after weight loss.** The two-step bariatric surgery cohort was comprised of 65 patients (66% women) with obesity class II or morbid obesity (minimum BMI > 38 kg/m2) who all completed a two-step bariatric surgery approach. This included a sleeve gastrectomy as the first step and laparoscopic Roux-en-Y gastric bypass as second step. Patients included in this study had a preoperative mean BMI of 54.7 ± 9.3 kg/m2 and an age of 45.3 ± 9.8 years. Before the second surgery, the patients mean BMI was 40.9 ± 7.3 kg/m2 with an mean age of 47.3 ± 9.9 years. On average, the patients lost 40.2 ± 21.2 kg between the two surgeries, and only individuals with a weight loss of more than five kilograms were included in the study. Parametric hypothesis testing (Welch’s one way ANOVA; Games-Howell post-hoc Test) were used and the pairwise *p*-values were corrected for multiple inference using the Hommel’s methods. **c) Validation of transcription factor gene expression upregulated in abdominal subcutaneous adipose tissue (OVAT) in insulin-sensitive vs. insulin resistant individuals.** The metabolically healthy versus unhealthy obese cohort (MHUO) comprises paired samples of omental visceral and abdominal subcutaneous adipose tissue from 31 insulin-sensitive patients (IS; 71% female; age: 38.8 ± 11.1 years old; BMI: 45.9 ± 6.9 kg/m²; FPG: 5.2 ± 0.2 mmol/l; FPI: 27.9 ± 13.5 pmol/l) and 42 insulin-resistant patients (IR; 71.43% female; age: 47.2 ± 7.7 years old; BMI: 47.3 ± 8.1 kg/m²; fasting plasma glucose (FPG): 5.7 ± 0.3 mmol/l; fasting plasma insulin (FPI): 113.7 ± 45.7 pmol/l). Parametric hypothesis testing (Welch’s one way ANOVA; Games-Howell post-hoc Test) were used and the pairwise *p*-values were corrected for multiple inference using the Hommel’s methods.

Supplementary Fig. S3a **Fig. S3: a) Validation of transcription factor gene expression upregulated in omental visceral adipose tissue (OVAT) in a cross-sectional validation cohort.** The human cross-sectional cohort comprises paired samples of OVAT and abdominal SAT from 1,480 individuals of the Leipzig Obesity Biobank (LOBB) including individuals with obesity (N = 1449) and without obesity (N = 31). Parametric hypothesis testing (Welch’s one way ANOVA; Games-Howell post-hoc Test) were used and the pairwise *p*-values were corrected for multiple inference using the Hommel’s methods.

Supplementary Fig. S3b **Fig. S3: b) Validation of transcription factor gene expression upregulated in omental visceral adipose tissue (OVAT) in a bariatric surgery cohort before and after weight loss.** The two-step bariatric surgery cohort was comprised of 65 patients (66% women) with obesity class II or morbid obesity (minimum BMI > 38 kg/m2) who all completed a two-step bariatric surgery approach. This included a sleeve gastrectomy as the first step and laparoscopic Roux-en-Y gastric bypass as second step. Patients included in this study had a preoperative mean BMI of 54.7 ± 9.3 kg/m2 and an age of 45.3 ± 9.8 years. Before the second surgery, the patients mean BMI was 40.9 ± 7.3 kg/m2 with an mean age of 47.3 ± 9.9 years. On average, the patients lost 40.2 ± 21.2 kg between the two surgeries, and only individuals with a weight loss of more than five kilograms were included in the study. Parametric hypothesis testing (Welch’s one way ANOVA; Games-Howell post-hoc Test) were used and the pairwise *p*-values were corrected for multiple inference using the Hommel’s methods.

Supplementary Fig. S3c **Fig. S3: c) Validation of transcription factor gene expression upregulated in omental visceral adipose tissue (OVAT) in insulin-sensitive vs. insulin resistant individuals.** The metabolically healthy versus unhealthy obese cohort (MHUO) comprises paired samples of omental visceral and abdominal subcutaneous adipose tissue from 31 insulin-sensitive patients (IS; 71% female; age: 38.8 ± 11.1 years old; BMI: 45.9 ± 6.9 kg/m²; FPG: 5.2 ± 0.2 mmol/l; FPI: 27.9 ± 13.5 pmol/l) and 42 insulin-resistant patients (IR; 71.43% female; age: 47.2 ± 7.7 years old; BMI: 47.3 ± 8.1 kg/m²; fasting plasma glucose (FPG): 5.7 ± 0.3 mmol/l; fasting plasma insulin (FPI): 113.7 ± 45.7 pmol/l). Parametric hypothesis testing (Welch’s one way ANOVA; Games-Howell post-hoc Test) were used and the pairwise *p*-values were corrected for multiple inference using the Hommel’s methods.

Supplementary Fig. S4 **Fig. S4: a) Volcano plot of differentially expressed genes (DEGs) between SAT and OVAT depots.** Points highlighted in red and blue show OVAT and SAT specific differential expression respectively (as defined by a *log*_*2*_(Fold Change) > 1 and FDR adjusted *p*-value < 0.05). **b)** Upset plot, showing actual number of differentially expressed genes (DEGs) overlapping between (SAT and OVAT based DEGs) versus (Subcutaneous and Visceral purified adipocytes based DEGs). **c and d)** Bar charts showing Enricher based analysis of the overlapping DEGs between adipose tissue and purified adipocytes (403 genes). **c)** Bar chart shows top enriched terms (for the input gene set of 403 genes) from “PhenGenI_Association_2021” gene set library. This enrichr library “The Phenotype-Genotype Integrator” (PheGenI), merges NHGRI genome-wide association study (GWAS) catalog data with several databases housed at the National Centre for Biotechnology Information (NCBI), including Gene, dbGaP, OMIM, eQTL and dbSNP. **d)** Bar chart shows top enriched terms (for the input gene set of 403 genes) from ““Human_Gene_Atlas” gene set library. This enrichr Library contains upregulated genes in human tissues from BioGPS. The top 10 enriched terms are displayed as -log_10_(*p*-value), with the actual *p*-value shown next to each term. An asterisk (∗) next to a *p*-value indicates the term also has a significant adjusted *p*-value (<0.05).

Supplementary Fig. S5 **Fig. S5: Volcano plot of all the non-differentially expressed genes in SAT and OVAT and overlapping with differentially accessible regions in OVAT (red) versus SAT (blue) or regions without differential accessibility (grey) in their promoter region (defined as ±1 kb of their TSS)**.

Supplementary Fig. S6 **Fig. S6: Gene expression data of genes upregulated in omental visceral adipose tissue (OVAT) in a cross-sectional validation cohort.** The human cross-sectional cohort comprises paired samples of OVAT and abdominal SAT from 1,480 individuals of the Leipzig Obesity Biobank (LOBB) including individuals with obesity (N = 1449) and without obesity (N = 31). Parametric hypothesis testing (Welch’s one way ANOVA; Games-Howell post-hoc Test) were used and the pairwise *p*-values were corrected for multiple inference using the Hommel’s methods.

Supplementary Fig. S7 **Fig. S7: Validation of genes linked to metabolic pathways and upregulated in subcutaneous adipose tissue in a cross-sectional validation cohort.** The human cross-sectional cohort comprises paired samples of OVAT and abdominal SAT from 1,480 individuals of the Leipzig Obesity Biobank (LOBB) including individuals with obesity (N = 1449) and without obesity (N = 31). Parametric hypothesis testing (Welch’s one way ANOVA; Games-Howell post-hoc Test) were used and the pairwise *p*-values were corrected for multiple inference using the Hommel’s methods.
