# Supplementary figures and images for: Chromatin landscape in paired human visceral and subcutaneous adipose tissue and its impact on clinical variables in obesity
Source: eBioMedicine. 2025 Mar 20;114:105653. doi: 10.1016/j.ebiom.2025.105653 (PMC11976249; doi:10.1016/j.ebiom.2025.105653)

Fig. S1

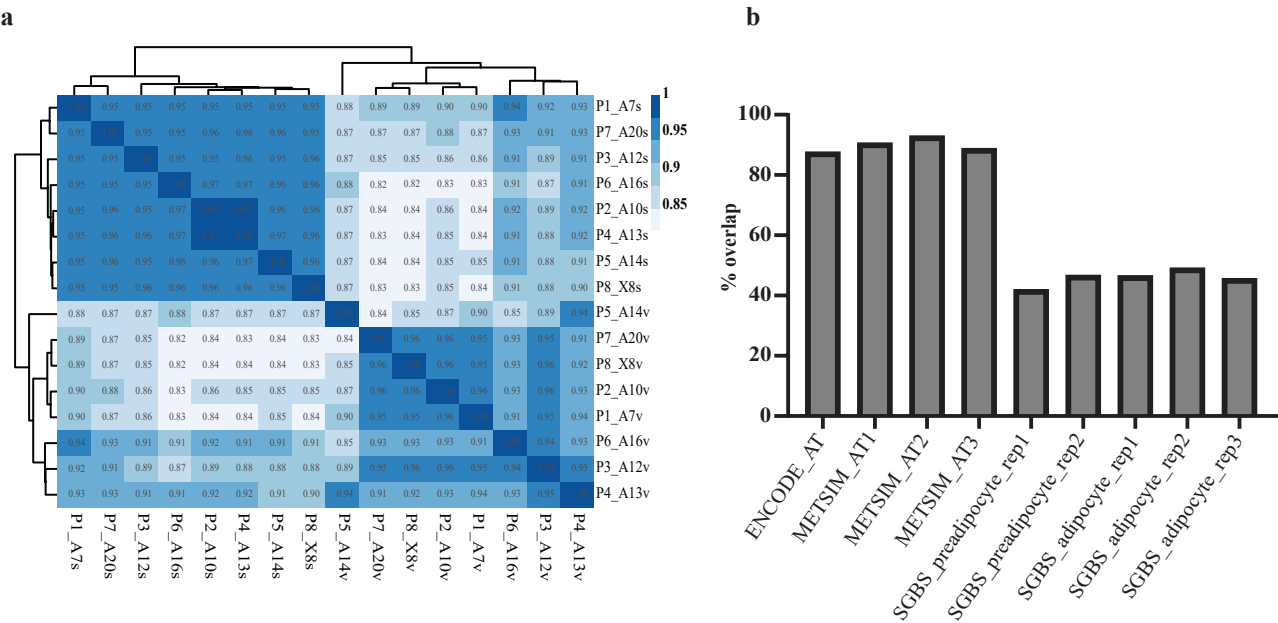

Supplement: Supplementary Fig. S1 — Fig. S1: a) ATAC-seq Data analysis: Clustered heatmap of Pearson correlation coefficients for SAT and OVAT ATAC-seq data across all biological replicates. Correlation is based on normalized log transformed read counts. b) Barplot showing percentage overlap of ATAC-seq peaks from different adipose tissue based datasets with our consensus peaks (166.129). [file mmc3.pdf]

Fig. S2

a

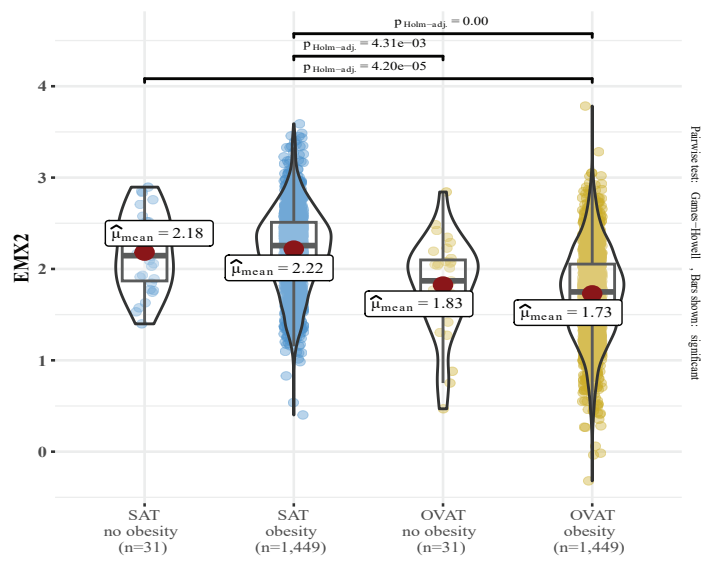

b

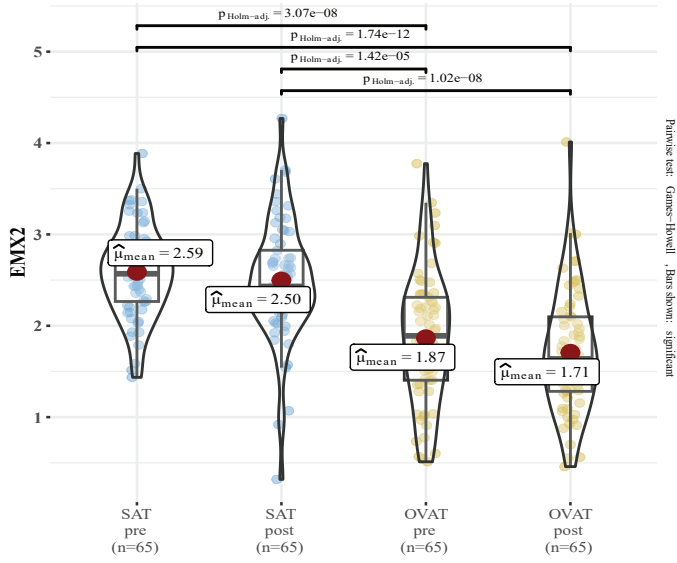

c

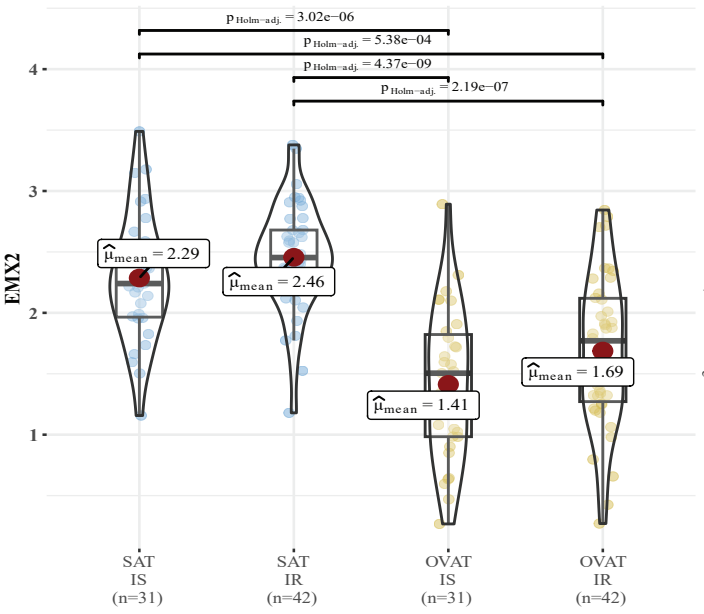

Supplement: Supplementary Fig. S2 — Fig. S2: a) Validation of transcription factor gene expression upregulated in abdominal subcutaneous adipose tissue (SAT) in a cross-sectional validation cohort. The human cross-sectional cohort comprises paired samples of OVAT and abdominal SAT from 1,480 individuals of the Leipzig Obesity Biobank (LOBB) including individuals with obesity (N = 1449) and without obesity (N = 31). Parametric hypothesis testing (Welch’s one way ANOVA; Games-Howell post-hoc Test) were used and the pairwise p-values were corrected for multiple inference using the Hommel’s methods. b) Validation of transcription factor gene expression upregulated in abdominal subcutaneous adipose tissue (OVAT) in a bariatric surgery cohort before and after weight loss. The two-step bariatric surgery cohort was comprised of 65 patients (66% women) with obesity class II or morbid obesity (minimum BMI > 38 kg/m2) who all completed a two-step bariatric surgery approach. This included a sleeve gastrectomy as the first step and laparoscopic Roux-en-Y gastric bypass as second step. Patients included in this study had a preoperative mean BMI of 54.7 ± 9.3 kg/m2 and an age of 45.3 ± 9.8 years. Before the second surgery, the patients mean BMI was 40.9 ± 7.3 kg/m2 with an mean age of 47.3 ± 9.9 years. On average, the patients lost 40.2 ± 21.2 kg between the two surgeries, and only individuals with a weight loss of more than five kilograms were included in the study. Parametric hypothesis testing (Welch’s one way ANOVA; Games-Howell post-hoc Test) were used and the pairwise p-values were corrected for multiple inference using the Hommel’s methods. c) Validation of transcription factor gene expression upregulated in abdominal subcutaneous adipose tissue (OVAT) in insulin-sensitive vs. insulin resistant individuals. The metabolically healthy versus unhealthy obese cohort (MHUO) comprises paired samples of omental visceral and abdominal subcutaneous adipose tissue from 31 insulin-sensitive patients (IS; 71% female; age [file mmc4.pdf]

Fig. S3a

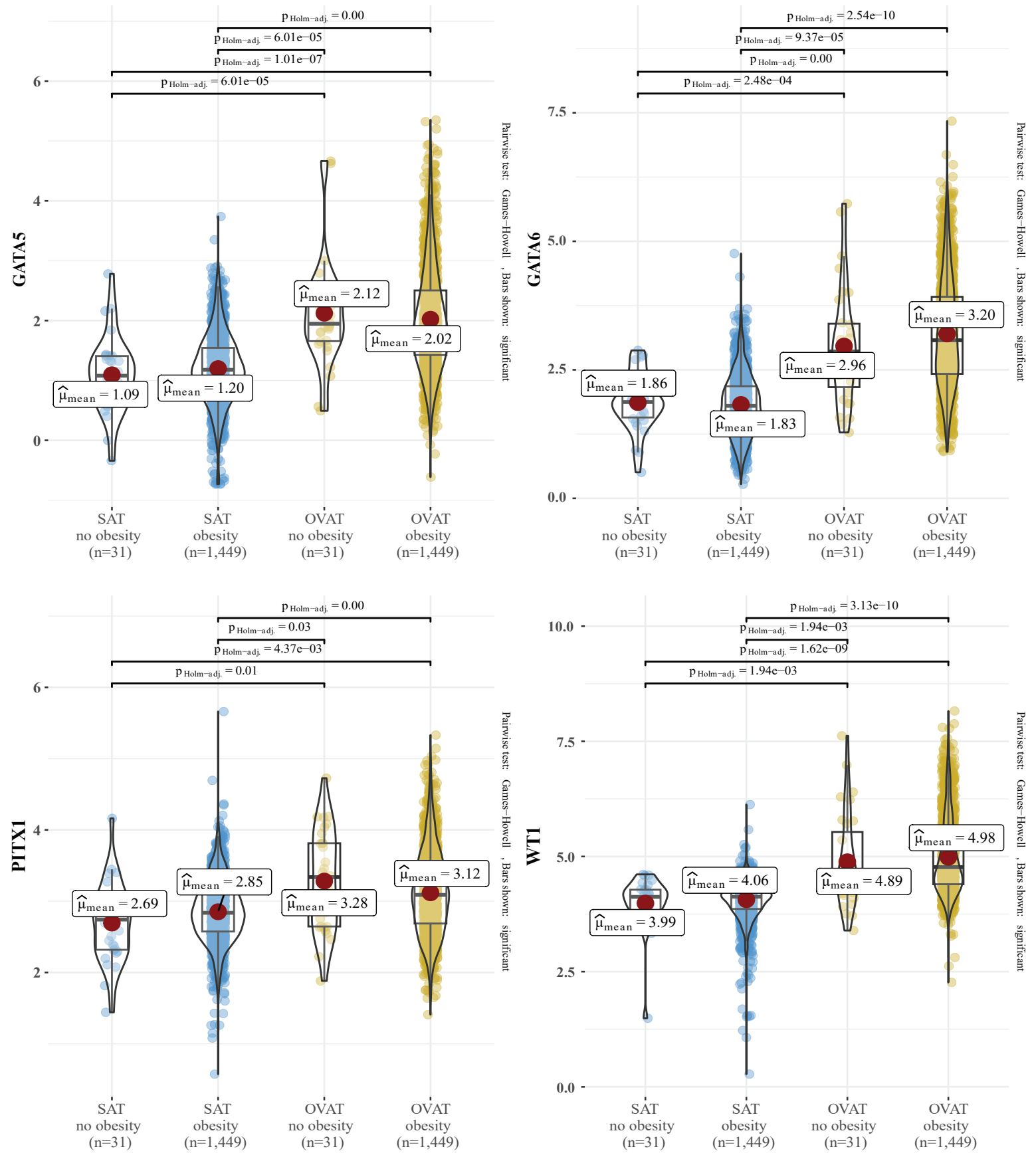

Supplement: Supplementary Fig. S3a — Fig. S3: a) Validation of transcription factor gene expression upregulated in omental visceral adipose tissue (OVAT) in a cross-sectional validation cohort. The human cross-sectional cohort comprises paired samples of OVAT and abdominal SAT from 1,480 individuals of the Leipzig Obesity Biobank (LOBB) including individuals with obesity (N = 1449) and without obesity (N = 31). Parametric hypothesis testing (Welch’s one way ANOVA; Games-Howell post-hoc Test) were used and the pairwise p-values were corrected for multiple inference using the Hommel’s methods. [file mmc5.pdf]

Fig. S3b

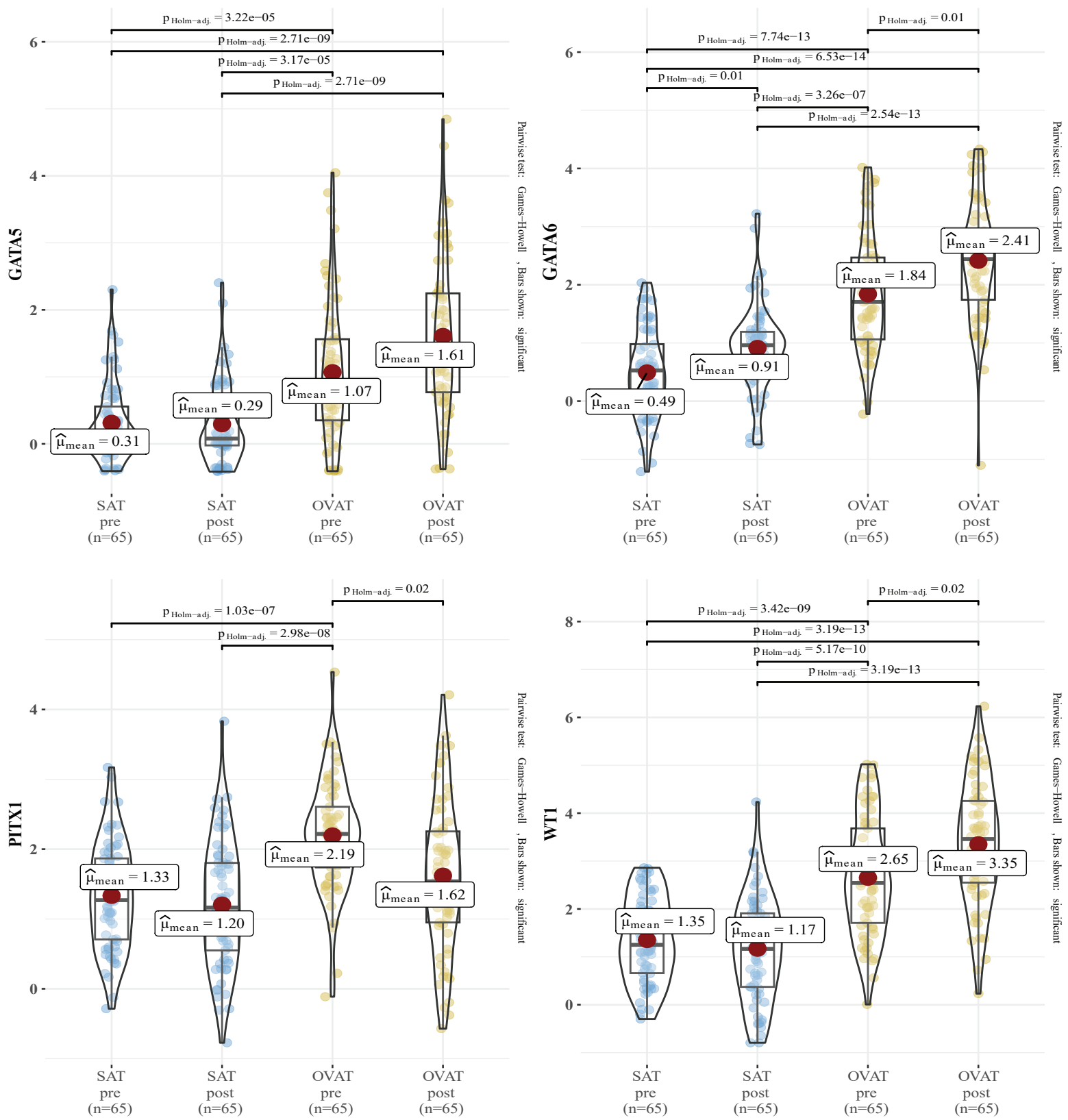

Supplement: Supplementary Fig. S3b — Fig. S3: b) Validation of transcription factor gene expression upregulated in omental visceral adipose tissue (OVAT) in a bariatric surgery cohort before and after weight loss. The two-step bariatric surgery cohort was comprised of 65 patients (66% women) with obesity class II or morbid obesity (minimum BMI > 38 kg/m2) who all completed a two-step bariatric surgery approach. This included a sleeve gastrectomy as the first step and laparoscopic Roux-en-Y gastric bypass as second step. Patients included in this study had a preoperative mean BMI of 54.7 ± 9.3 kg/m2 and an age of 45.3 ± 9.8 years. Before the second surgery, the patients mean BMI was 40.9 ± 7.3 kg/m2 with an mean age of 47.3 ± 9.9 years. On average, the patients lost 40.2 ± 21.2 kg between the two surgeries, and only individuals with a weight loss of more than five kilograms were included in the study. Parametric hypothesis testing (Welch’s one way ANOVA; Games-Howell post-hoc Test) were used and the pairwise p-values were corrected for multiple inference using the Hommel’s methods. [file mmc6.pdf]

Fig. S3c

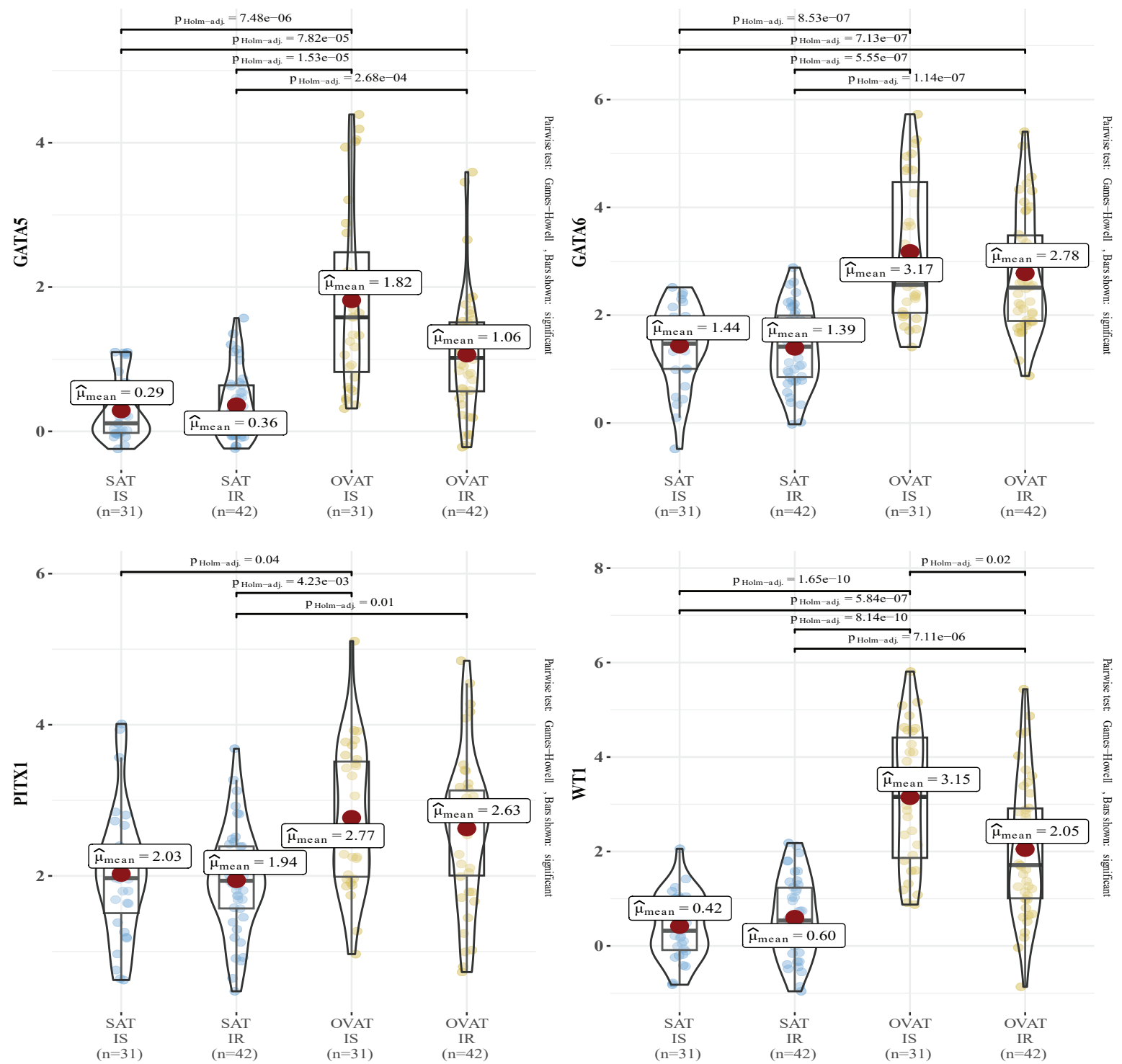

Supplement: Supplementary Fig. S3c — Fig. S3: c) Validation of transcription factor gene expression upregulated in omental visceral adipose tissue (OVAT) in insulin-sensitive vs. insulin resistant individuals. The metabolically healthy versus unhealthy obese cohort (MHUO) comprises paired samples of omental visceral and abdominal subcutaneous adipose tissue from 31 insulin-sensitive patients (IS; 71% female; age: 38.8 ± 11.1 years old; BMI: 45.9 ± 6.9 kg/m²; FPG: 5.2 ± 0.2 mmol/l; FPI: 27.9 ± 13.5 pmol/l) and 42 insulin-resistant patients (IR; 71.43% female; age: 47.2 ± 7.7 years old; BMI: 47.3 ± 8.1 kg/m²; fasting plasma glucose (FPG): 5.7 ± 0.3 mmol/l; fasting plasma insulin (FPI): 113.7 ± 45.7 pmol/l). Parametric hypothesis testing (Welch’s one way ANOVA; Games-Howell post-hoc Test) were used and the pairwise p-values were corrected for multiple inference using the Hommel’s methods. [file mmc7.pdf]

Fig. S4

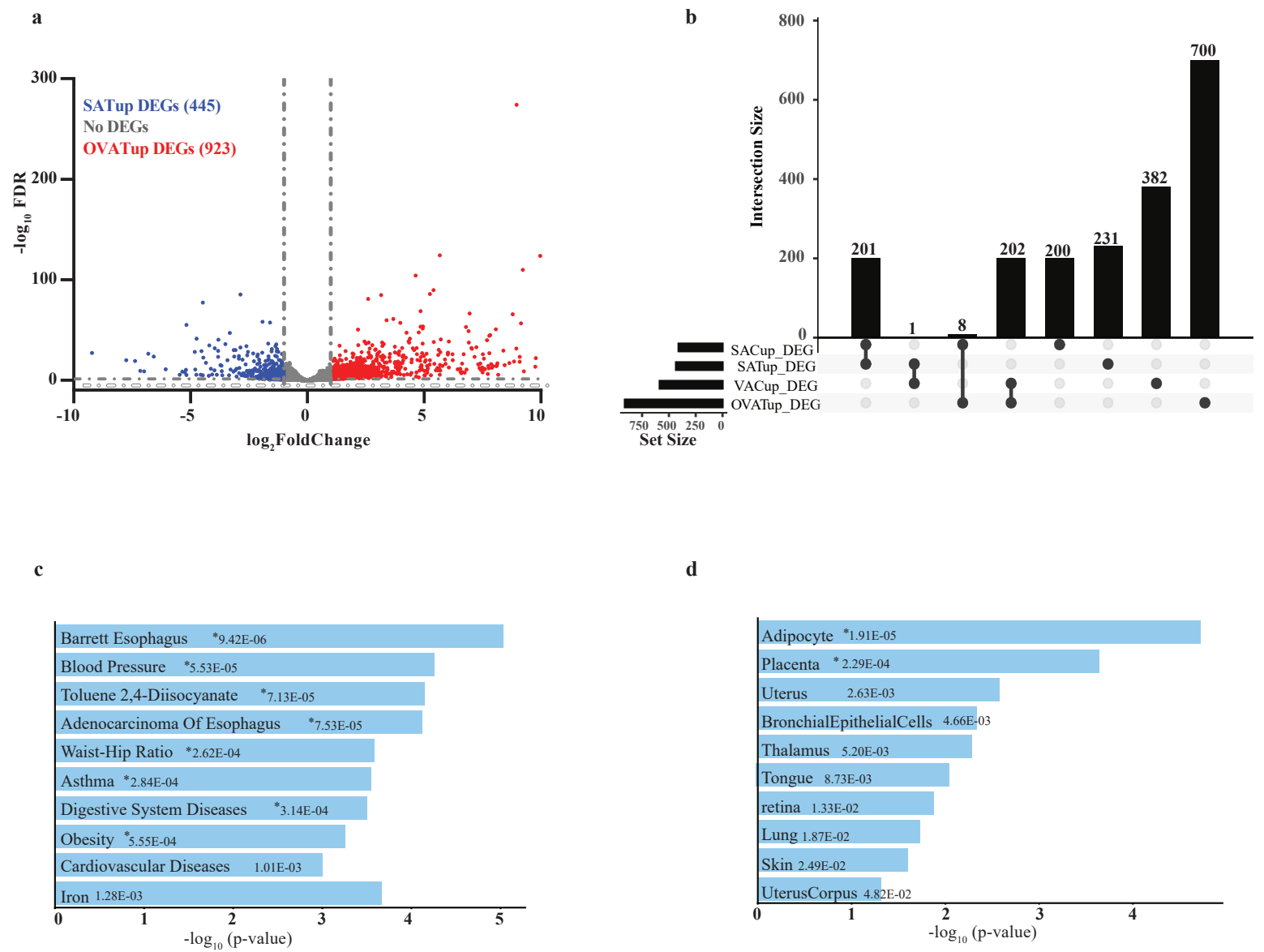

Supplement: Supplementary Fig. S4 — Fig. S4: a) Volcano plot of differentially expressed genes (DEGs) between SAT and OVAT depots. Points highlighted in red and blue show OVAT and SAT specific differential expression respectively (as defined by a log2(Fold Change) > 1 and FDR adjusted p-value < 0.05). b) Upset plot, showing actual number of differentially expressed genes (DEGs) overlapping between (SAT and OVAT based DEGs) versus (Subcutaneous and Visceral purified adipocytes based DEGs). c and d) Bar charts showing Enricher based analysis of the overlapping DEGs between adipose tissue and purified adipocytes (403 genes). c) Bar chart shows top enriched terms (for the input gene set of 403 genes) from “PhenGenI_Association_2021” gene set library. This enrichr library “The Phenotype-Genotype Integrator” (PheGenI), merges NHGRI genome-wide association study (GWAS) catalog data with several databases housed at the National Centre for Biotechnology Information (NCBI), including Gene, dbGaP, OMIM, eQTL and dbSNP. d) Bar chart shows top enriched terms (for the input gene set of 403 genes) from ““Human_Gene_Atlas” gene set library. This enrichr Library contains upregulated genes in human tissues from BioGPS. The top 10 enriched terms are displayed as -log10(p-value), with the actual p-value shown next to each term. An asterisk (∗) next to a p-value indicates the term also has a significant adjusted p-value (<0.05). [file mmc8.pdf]

Fig. S5

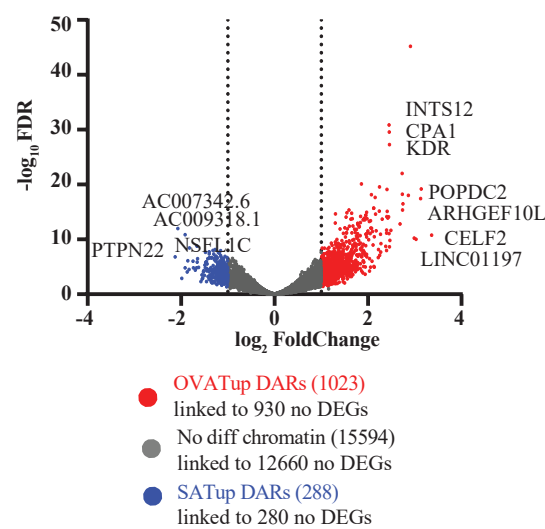

Supplement: Supplementary Fig. S5 — Fig. S5: Volcano plot of all the non-differentially expressed genes in SAT and OVAT and overlapping with differentially accessible regions in OVAT (red) versus SAT (blue) or regions without differential accessibility (grey) in their promoter region (defined as ±1 kb of their TSS). [file mmc9.pdf]

Fig. S6

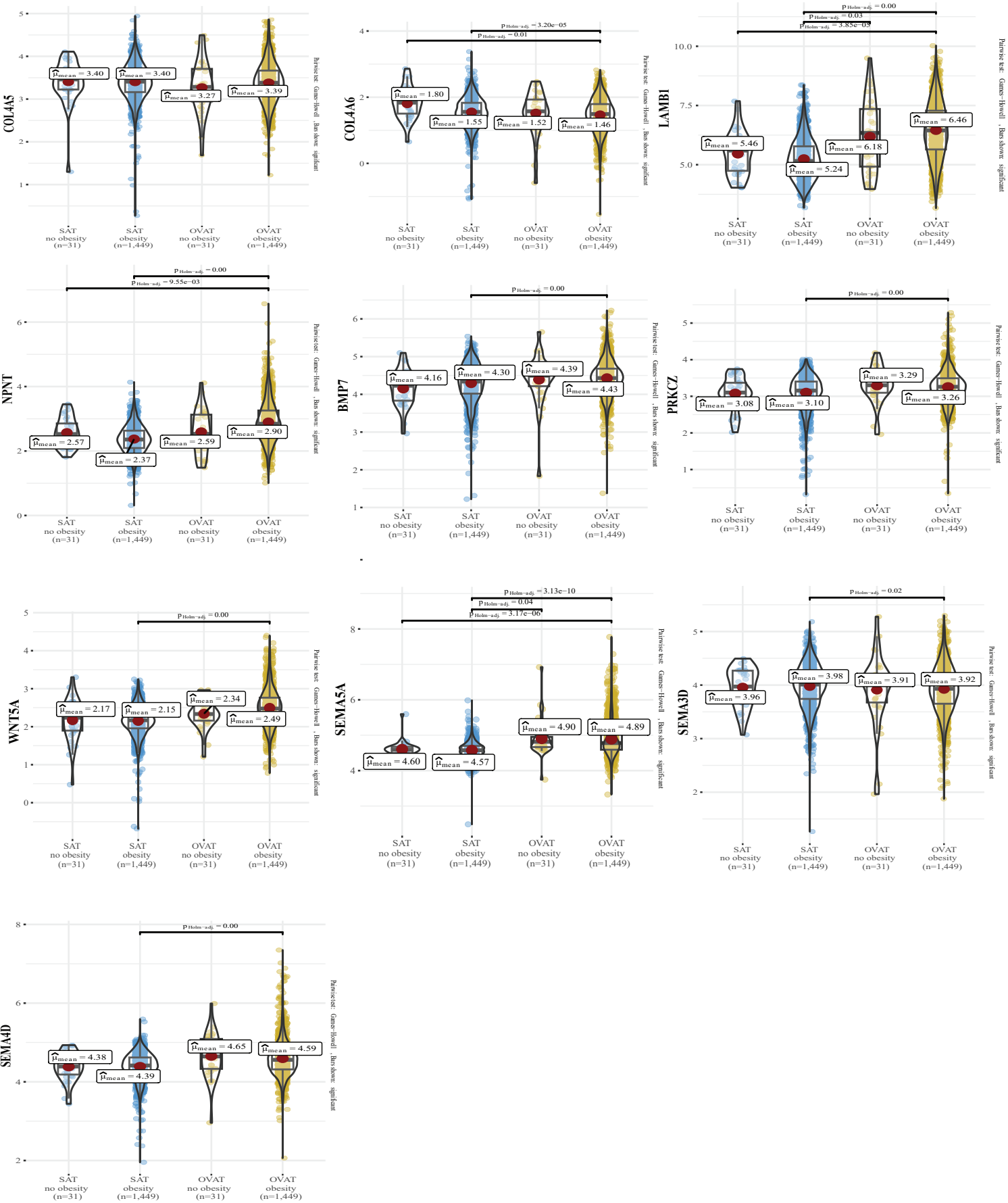

Supplement: Supplementary Fig. S6 — Fig. S6: Gene expression data of genes upregulated in omental visceral adipose tissue (OVAT) in a cross-sectional validation cohort. The human cross-sectional cohort comprises paired samples of OVAT and abdominal SAT from 1,480 individuals of the Leipzig Obesity Biobank (LOBB) including individuals with obesity (N = 1449) and without obesity (N = 31). Parametric hypothesis testing (Welch’s one way ANOVA; Games-Howell post-hoc Test) were used and the pairwise p-values were corrected for multiple inference using the Hommel’s methods. [file mmc10.pdf]

Fig. S7

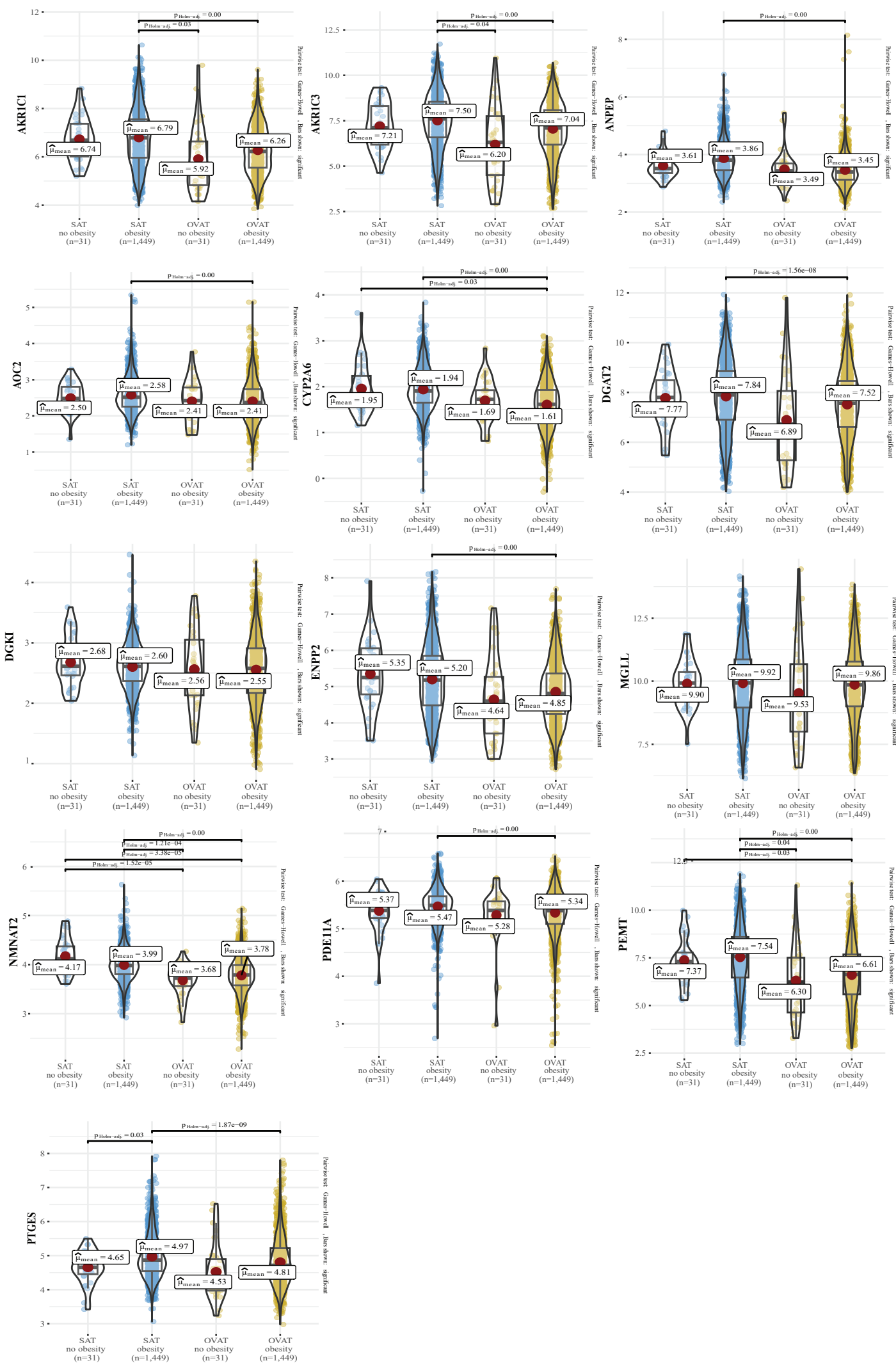

Supplement: Supplementary Fig. S7 — Fig. S7: Validation of genes linked to metabolic pathways and upregulated in subcutaneous adipose tissue in a cross-sectional validation cohort. The human cross-sectional cohort comprises paired samples of OVAT and abdominal SAT from 1,480 individuals of the Leipzig Obesity Biobank (LOBB) including individuals with obesity (N = 1449) and without obesity (N = 31). Parametric hypothesis testing (Welch’s one way ANOVA; Games-Howell post-hoc Test) were used and the pairwise p-values were corrected for multiple inference using the Hommel’s methods. [file mmc11.pdf]
